# Preparing for Life: Plasma Proteome Changes and Immune System Development During the First Week of Human Life

**DOI:** 10.3389/fimmu.2020.578505

**Published:** 2020-10-20

**Authors:** Tue Bjerg Bennike, Benoit Fatou, Asimenia Angelidou, Joann Diray-Arce, Reza Falsafi, Rebecca Ford, Erin E. Gill, Simon D. van Haren, Olubukola T. Idoko, Amy H. Lee, Rym Ben-Othman, William S. Pomat, Casey P. Shannon, Kinga K. Smolen, Scott J. Tebbutt, Al Ozonoff, Peter C. Richmond, Anita H. J. van den Biggelaar, Robert E. W. Hancock, Beate Kampmann, Tobias R. Kollmann, Ofer Levy, Hanno Steen

**Affiliations:** ^1^Department of Pathology, Boston Children’s Hospital, Boston, MA, United States; ^2^Precision Vaccines Program, Boston Children’s Hospital, Boston, MA, United States; ^3^Harvard Medical School, Boston, MA, United States; ^4^Department of Health Science and Technology, Aalborg University, Aalborg, Denmark; ^5^Department of Neonatology, Beth Israel Deaconess Medical Center, Boston, MA, United States; ^6^Department of Microbiology and Immunology, University of British Columbia, Vancouver, BC, Canada; ^7^Papua New Guinea Institute of Medical Research, Goroka, Papua New Guinea; ^8^Vaccines and Immunity Theme, Medical Research Council Unit, The Gambia at the London School of Hygiene and Tropical Medicine, Banjul, Gambia; ^9^Department of Molecular Biology and Biochemistry, Simon Fraser University, Burnaby, BC, Canada; ^10^Department of Pediatrics, University of British Columbia, and BC Children’s Hospital, Vancouver, BC, Canada; ^11^PROOF Centre of Excellence, Vancouver, BC, Canada; ^12^UBC Centre for Heart Lung Innovation, St. Paul’s Hospital, Vancouver, BC, Canada; ^13^Department of Medicine, Division of Respiratory Medicine, University of British Columbia, Vancouver, BC, Canada; ^14^Telethon Kids Institute, Perth, WA, Australia; ^15^Vaccine Centre, Faculty of Infectious and Tropical Diseases, London School of Hygiene and Tropical Medicine, London, United Kingdom; ^16^Department of Experimental Medicine, University of British Columbia, Vancouver, BC, Canada; ^17^Broad Institute of MIT & Harvard, Cambridge, MA, United States

**Keywords:** ontogeny, complement, innate immune system, immunoglobulin, proteomics, inhibitors, membrane attack complex (MAC), terminal complement complex (SC5b-9)

## Abstract

Neonates have heightened susceptibility to infections. The biological mechanisms are incompletely understood but thought to be related to age-specific adaptations in immunity due to resource constraints during immune system development and growth. We present here an extended analysis of our proteomics study of peripheral blood-plasma from a study of healthy full-term newborns delivered vaginally, collected at the day of birth and on day of life (DOL) 1, 3, or 7, to cover the first week of life. The plasma proteome was characterized by LC-MS using our established 96-well plate format plasma proteomics platform. We found increasing acute phase proteins and a reduction of respective inhibitors on DOL1. Focusing on the complement system, we found increased plasma concentrations of all major components of the classical complement pathway and the membrane attack complex (MAC) from birth onward, except C7 which seems to have near adult levels at birth. In contrast, components of the lectin and alternative complement pathways mainly decreased. A comparison to whole blood messenger RNA (mRNA) levels enabled characterization of mRNA and protein levels in parallel, and for 23 of the 30 monitored complement proteins, the whole blood transcript information by itself was not reflective of the plasma protein levels or dynamics during the first week of life. Analysis of immunoglobulin (Ig) mRNA and protein levels revealed that IgM levels and synthesis increased, while the plasma concentrations of maternally transferred IgG1-4 decreased in accordance with their *in vivo* half-lives. The neonatal plasma ratio of IgG1 to IgG2-4 was increased compared to adult values, demonstrating a highly efficient IgG1 transplacental transfer process. Partial compensation for maternal IgG degradation was achieved by endogenous synthesis of the IgG1 subtype which increased with DOL. The findings were validated in a geographically distinct cohort, demonstrating a consistent developmental trajectory of the newborn’s immune system over the first week of human life across continents. Our findings indicate that the classical complement pathway is central for newborn immunity and our approach to characterize the plasma proteome in parallel with the transcriptome will provide crucial insight in immune ontogeny and inform new approaches to prevent and treat diseases.

## Introduction

Bacteria and viruses that cause mild to no disease in adults can be life-threatening in newborns and infants (1). Neonatal infections cause 700,000 annual casualties, corresponding to 40% of deaths in children under 5 years of age (2). The biological mechanisms responsible for the early age-specific susceptibility are thought to be related to immune system development and age-specific adaptations in immunity due to resource constraints (1–3). This results in heightened vulnerability in early life where immune protection primarily relies on the innate immune system including leukocytes, cytokines, and the complement system, and the added protection from maternal factors including IgG antibodies transferred to the fetus over the placenta and IgA from breast milk (4–6). The complement system is a central element in the innate immune system and activation initiates several defense mechanisms, including enhancing circulating immunoglobulins, opsonization, immune cell recruitment, regulation of adaptive immunity, and the direct disruption of cell membranes (5).

The developmental trajectory of the immune system is increasingly recognized as a major determinant for overall health throughout life (7). However, our knowledge of the early life immune ontogeny and molecular mechanisms involved remains limited. We recently published the most comprehensive systems biology study of the first week of human life to date, using high-dimensional analytic platforms (7). Systems biology studies, including proteomics and transcriptomics, generate inherently comprehensive data that can be analyzed on many levels. Herein we present an extended analysis of the proteomics data of blood plasma from newborns using an improved bioinformatic pipeline. The high-dimensional molecular measurements together with unbiased analytic approaches, enable a deep data-driven analysis. The aim was to identify and characterize molecular networks and signatures related to immune system changes during the first week of life. Given the importance for newborn immunity, we focused our analysis on the complement system and maternally transferred antibodies.

This deep data analysis has greatly expanded on our original findings, indicating a highly dynamic biological state during the first week of life, compared to a relatively steady state in healthy adults (8). Findings in our study’s main cohort from West Africa (The Gambia) were validated in an independent cohort from Australasia (Papua New Guinea, PNG). Even though dramatic changes occur in the first week of life, ontogeny follows a robust trajectory that is consistent even in geographically and ethnically distinct populations.

## Materials and Methods

### Study Cohort and Ethics

Thirty and nineteen healthy, term newborns were enrolled at the Medical Research Council (MRC) Unit The Gambia at London School of Hygiene and Tropical Medicine and at the Institute for Medical Research (IMR) in Goroka, Papua New Guinea in accordance, with a local Ethics Committee-approved protocol (MRC SCC 1436 and IMR IRB#1515 and MRAC #16.14). A detailed description of the protocol has been published (9).

**Figure 1:**
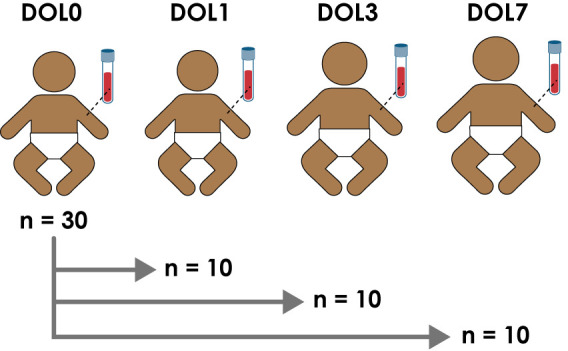
Study design and number of enrolled newborns in the main cohort enrolled in The Gambia.

Following informed consent, mothers were screened for HIV-I and -II and hepatitis B. Positivity for either virus represented an exclusion criterion. Inclusion criteria were a healthy appearing infant as determined by physical examination, born by vaginal delivery at gestational age of >36 weeks, with a 5-min Apgar score > 8, and a birth weight of >2.5 kg. Peripheral blood samples were obtained from all infants on the day of birth (DOL0) and then again either at DOL1, DOL3, or DOL7 to limit phlebotomy to a maximum of twice in the first week of life (**Figure 1** and **Supplementary Figure 1**). Peripheral venous blood was drawn from infants *via* sterile venipuncture directly into heparinized collection tubes [Becton Dickinson (BD) Biosciences; San Jose, CA, USA]. Aliquots (200 μl) were immediately placed in RNA-later (Ambion Thermo Fisher; Waltham, MA, USA) for RNAseq analysis with the remaining blood kept in the collection tubes at room temperature. Within 4 h, the whole blood was centrifuged on site at 500 × g for 10 min at room temp and the plasma was stored at −80°C. All samples were shipped on dry ice (World Courier; New Hyde Park, NY, USA).

Plasma cytokine profiles were determined using a custom designed multi-analyte Cytokine Human Magnetic Panel Bead Array (Invitrogen/Life Technologies; Carlsbad, CA). Metabolite profiles were determined by metabolomics (Metabolon, Durham, NC, USA). Data was downloaded from the original publication (7) and re-analyzed.

### Proteomics Sample Preparation

For the original study (7), plasma samples from newborns were prepared for proteome analysis using the in-house developed plasma and serum proteomics workflow, based on the MStern blotting sample processing and trypsinization protocol (10, 11). To this end, 5 µL plasma were diluted in 100 µL sample buffer (8 M urea in TRIS-HCl, pH 8.5). Protein disulfide bonds were reduced with dithiothreitol (10 mM final concentration) and alkylated with iodoacetamide (50 mM final concentration). An aliquot with 10 µg protein was transferred to a 96 well plate with a polyvinylidene fluoride (PVDF) membrane bottom (MSIPS4510, Millipore, MA, USA). Protein digestion was performed with sequencing-grade modified trypsin (V5111, Promega, Madison, WI, USA) at a nominal protease to protein ratio of 1:25 w/w. After incubation for 2 h at 37°C, the peptides were eluted and concentrated to dryness in a vacuum centrifuge. To monitor retention time stability and system performance, iRT peptides (Biognosys, Schlieren, Switzerland) were spiked into all samples.

### Mass Spectrometry Analysis

The samples were analyzed using a nanoLC system (Eksigent, Dublin, CA) equipped with a LCchip system (cHiPLC nanoflex, Eksigent, CA, USA) coupled online to a Q Exactive Mass Spectrometer (Thermo Scientific, Bremen, Germany). From each sample, 0.2 µg peptide material was separated using a linear gradient from 93% solvent A (0.1% formic acid in water), 7% solvent B (0.1% formic acid in acetonitrile) which was increased to 32% solvent B over 60 min. The mass spectrometer was operated in data-dependent mode, selecting up to the 12 most intense precursors for fragmentation from each precursor scan.

### Proteomics Data Analysis

Unbiased data-driven analytical approaches have the advantage that the data can be repeatedly interrogated based on varying starting hypotheses. Using the proteomics raw data from the original study (7), we performed a label-free protein quantitation (LFQ) analysis in the newest version of MaxQuant (v 1.6.2.5) using standard settings with quantitation by razor (protein-group shared) and unique peptides and LFQ normalization (12). Proteins were identified using the built-in Andromeda search engine and an updated reviewed UniProt Human Reference Proteome (13). Standard search settings were employed with matching between runs on and the following abundant modifications: max three tryptic missed cleavages, methionine oxidation as variable modification, and cysteine carbamidomethylation as fixed modification (11). The revert decoy search strategy in MaxQuant was used to filter all reported proteins and peptides to <1% false discovery rate (FDR) (14).

The lists of identified protein groups (henceforth referred to as proteins) and relative protein LFQ quantities were imported into R using Rstudio (15, 16), prior to application of an analytical workflow beyond that commonly applied to proteomics. The following additional filtering criteria were applied for quantifiable proteins: removal of i) proteins, which are commonly introduced during the handling, preparation and processing of the samples, tagged as likely contaminants by MaxQuant (e.g., keratins, trypsin, etc.), ii) proteins only identified by peptides containing variable modifications, and iii) proteins that were quantifiable in less than 50% of DOL0 and DOL1, 3, or 7 study participants. The strict filtering strategy enabled us to avoid the need to impute missing datapoints, and only use actual data, except for conducting principal component analysis (PCA) where missing values were replaced with values from a Gaussian distribution (q = 0.01, tune.sigma = 0.3) to simulate signals from low abundant proteins (17). ComBat R-package was used to correct for batch effects for samples run on different LC-MS columns and MStern plates (18). Quantro R-package was used to analyze for global differences in the protein abundances between the different DOLs (10,000 simulations), which could point to methodological problems (19). Additionally, ggplot2 was used for visualizations, MixOmics for analysis, and dplyr for data matrix formatting (20–22). All data is publicly available and can be accessed through this publication. The mass spectrometry RAW data and search results have been deposited to the ProteomeXchange consortium *via* the PRIDE partner repository and are available with the data set identifier PXD019817, as well as archived on ImmPort (https://immport.niaid.nih.gov/home) under accession numbers SDY1256 and SDY1412 (23, 24).

### Differential Protein Expression Analysis and Bioinformatics

We fitted a linear mixed-effects regression model of intensity, with fixed effects of DOL, sex, LC column/MStern plate, and a random participant effect, using the lmer function from the lme4 R package (25). Proteins that were quantifiable in less than 50% of DOL0 and DOL1, 3, or 7 study participants were removed, and remaining missing values were not included in the analysis. P-values and fold changes were calculated DOL-wise using the paired experimental design, with a null-model without DOL using the ANOVA-function, and we controlled the FDR by applying the Benjamini–Hochberg correction method (26). Proteins were considered to be significantly differentially regulated at 5% FDR and +/− 0.2 log2 foldchange. Additionally, we performed a missing-value analysis based on Fisher’s exact testing on the data matrix prior to filtering, as the strict valid value filtering scheme would have removed DOL-unique proteins. Significant proteins were further analyzed using WebGestaltR for pathway analysis, StringDB for obtaining protein-protein interaction networks, and Cytoscape for visualization (27–29). Boxplot center lines show the medians, box limits indicate the 25^th^ and 75^th^ percentiles, whiskers extend 1.5 times the interquartile range from the 25^th^ and 75^th^ percentiles, outliers are represented by dots. FDR adjusted p-values (q-values) from the statistical models are reported on the boxplots, unless otherwise stated.

### Immunoglobulin G Subclass Ratio Analysis

The relative inter-abundances of the IgG subclasses were estimated using the intensity-based absolute quantitation (iBAQ) values from MaxQuant, calculated from unique peptide abundances only (uploaded to the PRIDE repository PXD019817). To enable a comparison to the adult state, we used data from a previously published proteomics study of plasma from 30 healthy adult Danes (10). To compensate for the different experimental designs, we normalized the IgG ratios to IgG2 (of note, normalization to IgG3 yielded similar results) in each dataset. Significantly different Ig-ratios were calculated using two-sample t-tests.

### RNA Sample Preparation and Sequencing

For RNA sequencing, total RNA was extracted from whole blood using the RiboPure RNA Purification Kit. Quantification and quality assessment of total RNA was performed on an Agilent 2100 Bioanalyzer. Samples with sufficiently high RNA integrity number were considered for sequencing. Poly-adenylated RNA was captured using the NEBNext Poly(A) mRNA Magnetic Isolation Module. Strand-specific cDNA libraries were generated from poly-adenylated RNA using the KAPA Stranded RNA-Seq Library Preparation Kit and sequenced on a HiSeq 2500 (Illumina; San Diego, CA, USA). Sequence quality was assessed using FastQC and MultiQC1.8.1 (30). The FASTQ sequence reads were aligned to the human genome (Ensembl GRCh38.98) using STAR v2.7 and mapped to Ensembl GRCh38 transcripts (31). Read-counts were generated using htseq-count (HTSeq 0.11.2-1) (32). Data processing and subsequent differential gene expression (DGE) analyses were performed using the latest versions of R and DESeq2 using the Wald statistics and paired analysis (33). Genes with very low counts (with less than 10 counts in nine or more samples, or the smallest number of biological replicates within each treatment group) and globin transcripts were filtered out prior to DGE analysis.

## Results and Discussion

### Enrolled Participants

Blood samples were collected employing a sample-sparing protocol, which enabled characterization of the plasma proteome, plasma metabolome, cytokine/chemokine profile, whole blood transcriptome, and single cell immunophenotype in the same sample (7). We utilized a paired study design with a blood sample collected from all newborns on the day of life (DOL) 0, and a matching follow-up sample either at DOL1, 3, or 7 (**Figure 1**). The main cohort included 60 samples collected from 30 newborns in The Gambia/West Africa and the smaller validation cohort comprised 38 samples collected from 19 newborns in Papua New Guinea (PNG)/Australasia using a similar experimental design (**Supplementary Figure 1**).

Having access to peripheral blood samples rather than cord blood for the DOL0 sample, provided each subject with an ideal specific baseline-sample. This would not have been the case with cord-blood as differences between peripheral and cord-blood have been reported (34). Findings in this study are limited to healthy babies born by vaginal delivery.

### Quantifiable Proteins in Newborn Blood Plasma Cluster by Day of Life

We analyzed plasma samples using our published plasma proteomics workflow in a 96-well plate format, and identified 382 plasma proteins (FDR<1%), of which 197 passed our criteria for quantifiable proteins (10, 11). These numbers are comparable to those reported in similar plasma- and serum studies (10, 35, 36). Depletion of high-abundant proteins prior to analysis, such as albumin, complement component proteins, and immunoglobulins, could increase the number of monitored plasma proteins (10), but could also interfere with the abundance of other proteins, since e.g., albumin functions as a protein carrier (37). Therefore, to ensure the highest quality of data, we chose not to deplete the high-abundant plasma-proteins prior to proteomics analysis, which also enables us to characterize the levels of these, many of which are involved in modulating the immune system, and were as such relevant to our study.

**Figure 2:**
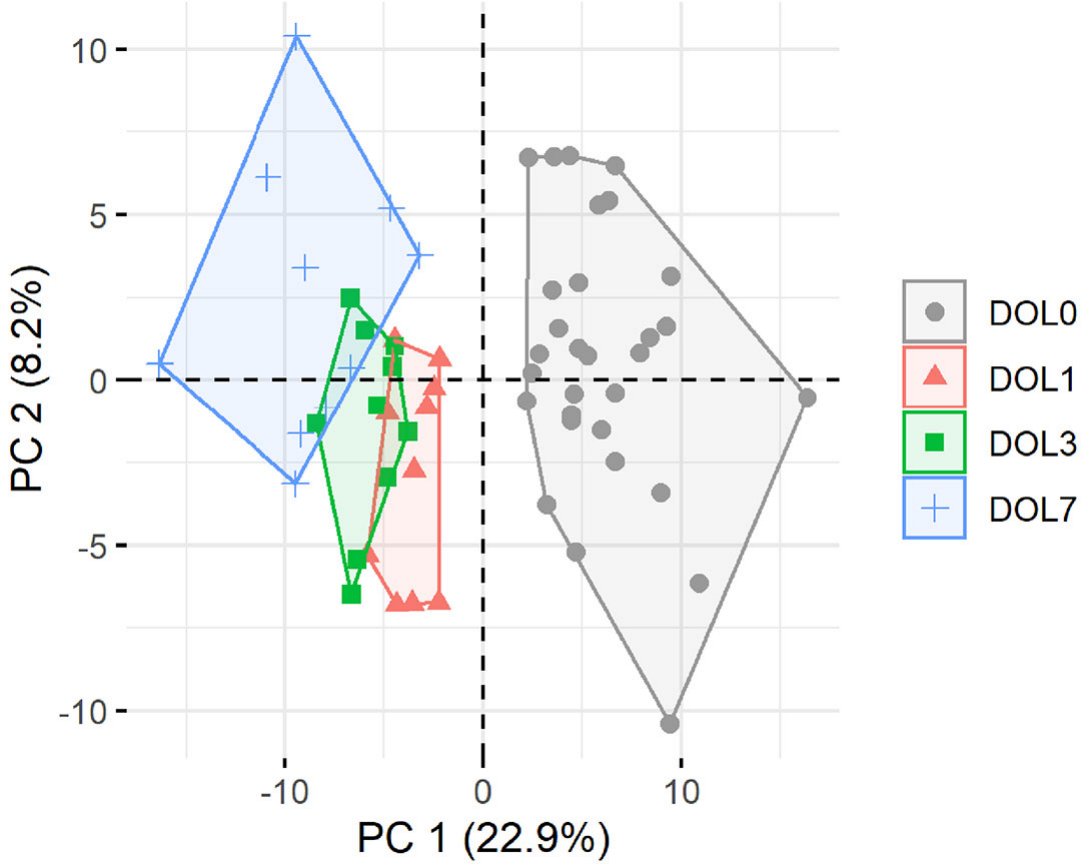
Principal component analysis (PCA) plot of all quantifiable proteins separates samples by day of life (DOL). PC, principal component. Explained variance given in percentages.

Principal component analysis (PCA) of quantifiable proteins in newborn blood plasma revealed consistent changes over the first week in both the main cohort (**Figure 2**) and the validation cohort (**Supplementary Figure 2**), demonstrating a common developmental trajectory of the newborn plasma proteome during the first week of life across continents.

### The Number of Changed Plasma-Proteins Increases With Age

**Figure 3:**
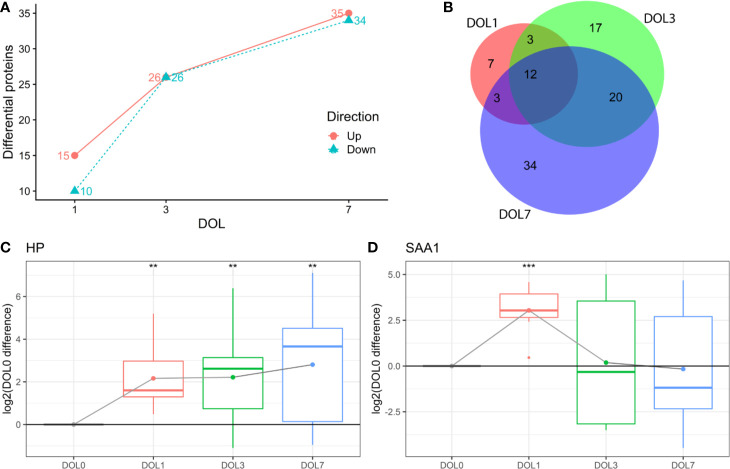
Differentially abundant plasma proteins (q-value < 0.05) compared to day of life (DOL) 0. **(A)** We identified a robust trajectory of differentially expressed proteins over the first week of life. **(B)** Overlap of regulated proteins. Protein regulations of **(C)** Haptoglobin (HP). **(D)** Serum amyloid A1 (SAA1) normalized to DOL0, with mean abundance difference indicated by dots connected with a line. q-value * <0.05, **: <0.01, ***: <0.001.

This study aimed to identify differentiating proteins over the first week of life. For this purpose, we performed a linear regression analysis of the protein abundance data. Our statistical model allowed us to identify an increasing number of proteins that showed statistically significant DOL-dependent abundance differences (**Figures 3A, B**, **Supplementary Tables 1–3**). A similar trajectory was seen in the validation cohort (**Supplementary Figures 3**-**5**, **Supplementary Tables 4–6**).

The use of linear regression analysis yielded increased sensitivity compared to analysis using t-tests, as evidenced by identification of twice the number of differentiating proteins (7), likely by achieving higher statistical power (38, 39).

The proteins with the largest abundance increase at DOL1 compared to DOL0 were the acute phase proteins haptoglobin (HP) and serum amyloid A-1 (SAA1) (**Figures 3C, D**). SAA2 was also increased but did not pass multiple hypothesis testing (q-value: 0.11, p-value 0.03) (**Supplementary Table 7**). HP, SAA1, and SAA2 were the top three most increased proteins in the validation cohort at DOL1 when disregarding statistical significance as the number of samples in the validation cohort was smaller (**Supplementary Table 8**). This demonstrates a consistent observation across geographically distinct cohorts and experiments.

**Figure 4:**
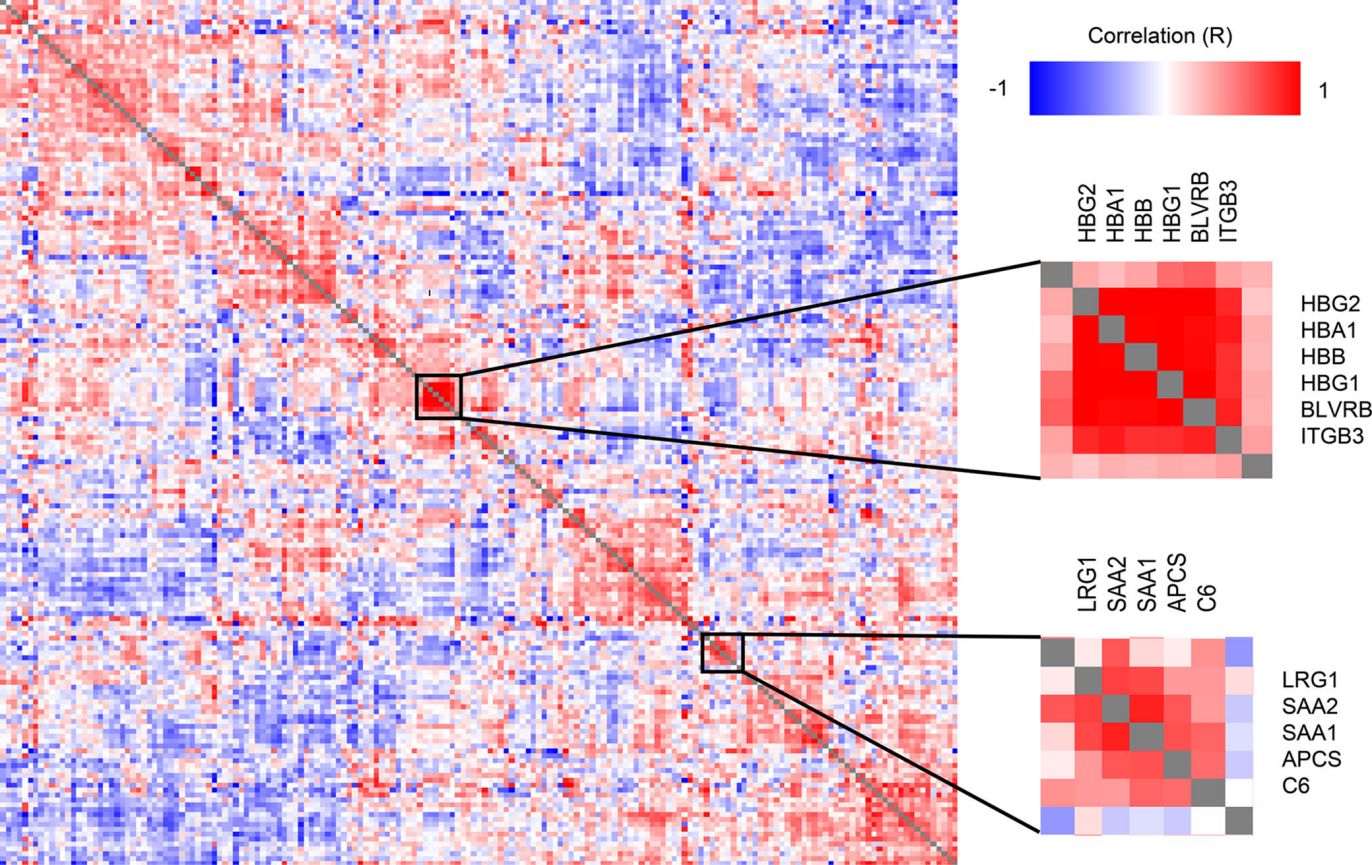
Clustered Pearson’s protein-protein correlation matrix of protein changes during the first week of life, of all quantifiable proteins (on the x- and y-axis) which allows for identifying proteins with similar trajectories across all samples. Several clusters of correlating proteins were identified, including a cluster centered around hemoglobin and an acute phase response cluster including SAA1 and SAA2.

A correlation analysis of all protein level change relative to DOL0 (**Figure 4**) revealed several clusters of proteins with similar trajectories, including a hemoglobin (Hb) cluster and an acute phase response cluster with SAA1, SAA2, and complement component C6. The expression of the various globin genes is strictly balanced and coordinated, and the positive correlation of the Hb subunits is expected and validates the applied methodology (40).

### Low Levels of Haptoglobin in the Hours After Birth

Several studies have failed to consistently measure HP in neonates and in particular at birth when concentrations are low, likely because of insufficient assay sensitivity (41). The reliable identification of HP at DOL0 demonstrates the high sensitivity of the applied methodology. The finding of increased HP after birth is in agreement with previous studies, where a significant increase in HP was measured at DOL2-4 compared to DOL0 (41), and in adults compared to neonates and children (42). Our study demonstrates that the increased HP can be measured as early as 24 h after birth.

The main physiological function of HP is to sequester free (extracellular) hemoglobin (Hb) released from destroyed red blood cells (43). The HP-Hb complex is quickly cleared from the circulation through monocytes and tissue macrophages *via* CD163 receptors, and degraded in lysosomes rather than being recycled which can lead to HP depletion (44). The gradual increase of HP at DOL1, 3, and 7 relative to DOL0, may reflect a physiologic rise of HP in neonatal plasma levels in these healthy newborn cohorts, preceding the need for clearance of the fetal hemoglobin HbF (α_2_γ_2_) from the neonatal circulation as it is gradually replaced by the adult type HbA (α_2_β_2_) over the first 6 months of life.

By binding excessive free Hb, HP exerts anti-inflammatory and anti-oxidant effects (43). Newborns and infants with a bacterial infection have increased HP-levels, and HP appears to regulate some adverse effects of inflammation (41) but the role of HP in neonatal sepsis is not yet fully understood. A study with a higher time-resolution could provide additional insights into the perinatal physiologic fluctuations of HP.

### Acute Phase Response Proteins Transiently Increase on Day of Life 1

SAA1 is an acute phase protein with antimicrobial activity (45) mainly produced in response to infections (46), and was significantly increased at DOL1 and returned to DOL0 levels at DOL3 and DOL7.

SAA1 is mainly synthesized in the liver, but can also be synthesized by stimulated monocytes and monocyte-derived macrophages (47). We were unable to identify the corresponding mRNA in the whole blood, which indicated that during the first week of life SAA synthesis might be limited to the hepatocytes rather than stimulated immune cells in circulation.

Increased SAA1-levels could indicate the presence of an acute phase response at DOL1. To identify proteins with a similar trajectory, we performed a correlation analysis of SAA1 against all proteins (**Table 1**). Most proteins correlating positively with SAA1 were involved in the acute phase response. The protein with the most negative correlation to SAA1, alpha-2-macroglobulin (A2M), is involved in inhibiting the complement system response. Additionally, clusterin (CLU), another central inhibitor of the complement cascades, correlated negatively with SAA1.

**Table 1:** Proteins with abundance differences significantly correlating (p-value < 0.05) with serum amyloid A-1 protein (SAA1) demonstrated an acute-phase response at DOL1.

R	P-value	Protein name	Gene name	Description	UPID
1.000	<2.2E−16	Serum amyloid A-1 protein	SAA1	Acute-phase response	P0DJI8
0.821	5.78E−04	Serum amyloid A-2 protein	SAA2	Acute-phase response	P0DJI9
0.672	3.16E−03	Leucine-rich alpha-2-glycoprotein	LRG1	Brown fat cell differentiation/bacterial response	P02750
0.642	3.06E−04	Serum amyloid P-component	APCS	Acute-phase response	P02743
0.587	1.28E−03	Fibrinogen alpha chain	FGA	Blood clotting/associated with infection	P02671
0.566	2.10E−03	Complement component C6	C6	Complement activation	P13671
0.512	6.27E−03	Alpha-1-antichymotrypsin	SERPINA3	Acute-phase response	P01011
0.459	1.60E−02	Haptoglobin	HP	Acute-phase response	P00738
0.413	3.21E−02	Alpha-1-acid glycoprotein 1	ORM1	Acute-phase response	P02763
0.405	3.59E−02	Fibrinogen beta chain	FGB	Blood clotting/associated with infection	P02675
0.389	4.51E−02	Neutrophil defensin 3	DEFA3	Antibacterial activities	P59666
−0.389	4.48E−02	Complement C1q subunit C	C1QC	Complement system	P02747
−0.423	2.81E−02	Plasma kallikrein	KLKB1	Convert prorenin into renin	P03952
−0.450	3.14E−02	Flavin reductase (NADPH)	BLVRB	Oxidoreductase	P30043
−0.458	1.63E−02	Clusterin	CLU	Inhibitor of complement	P10909
−0.484	1.05E−02	Inter-alpha-trypsin inhibitor heavy chain H2	ITIH2	Protease inhibitor	P19823
−0.509	6.73E−03	Apolipoprotein M	APOM	Cholesterol homeostasis	O95445
−0.751	6.41E−06	Alpha-2-macroglobulin	A2M	Inhibitor of complement	P01023

Altogether these findings demonstrate the presence of an acute-phase response 24 h after birth, driven by an increase of several acute-phase proteins and a simultaneous reduction of inhibitory proteins. Our findings are in line with a previous publication, where SAA1 levels in newborns in Sweden were increased at DOL1 and subsequently decreased at DOL2 and DOL3 (48), and an acute phase response was evident shortly after birth (49, 50). Our study further expands on the molecular mediators of the acute phase response, demonstrating the involvement of the complement system and simultaneous reduction of inhibitors. The relatively short *in vivo* half-life of SAA1 (51, 52) in the 90-min range makes it unlikely that the increase on DOL1 was directly caused by immune cell activation during vaginal delivery. This is supported by the lack of corresponding whole blood mRNA, indicating that hepatocytes rather than activated immune cells are the likely point of synthesis. Interleukin-6 (IL-6) is an inducer of hepatic acute phase protein synthesis (53), and of adrenal gland production of cortisol, a metabolite that enhances the acute phase response in part *via* increasing IL-6 receptor expression on hepatocytes (18). To further characterize molecular drivers of the acute phase response in early life, we investigated plasma IL-6 and cortisol concentrations, as determined by multi-plex assay and metabolomics, respectively. Likely reflecting recent labor (50), both IL-6 and cortisol levels were highest at DOL0 (7) (**Supplementary Table 9**), and likely initiate the subsequent physiologic acute phase response of the newborn (18, 50). Altered neonatal blood circulation and increased oxygen levels, as well as decreasing levels of circulating maternal factors in the newborn may also contribute to these early proteomic shifts. Speculatively, this first newborn inflammatory acute phase response could be a means of immune system development activation, in response to the extrauterine transition and anticipation of encounters with various microorganisms.

### Complement System Development Is Central Over the First Week of Life

**Figure 5:**
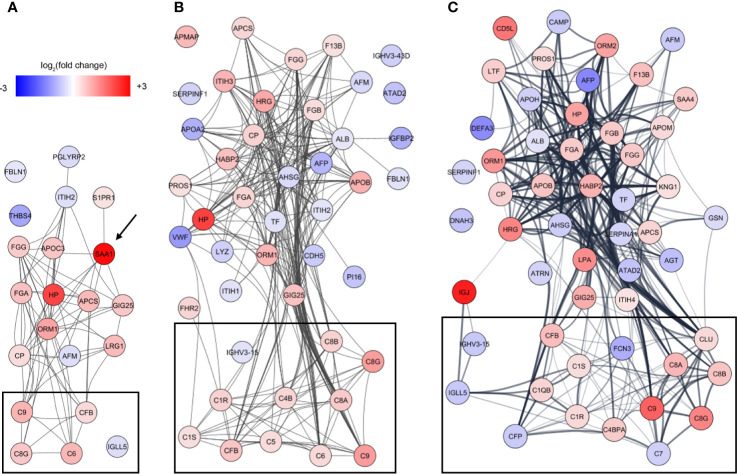
Analysis of protein-protein interactions of the differentiating proteins at **(A)** DOL1, **(B)** DOL3, and **(C)** DOL7 compared to DOL0 (at birth). SAA1 which was regulated at DOL1 only is indicated with an arrow, and lines indicate interacting proteins. Proteins tagged as complement system in Gene Ontology are indicated with black boxes without further curation.

To identify underlying biological themes in addition to the early acute phase response, we performed a protein-protein interaction analysis of the increasing number of proteins showing ontogenic changes during the first week of life. The analysis revealed that many of the differentiating proteins were functionally related, and especially a cluster of proteins involved in the complement system increased with DOL (**Figure 5**, boxed area).

**Figure 6:**
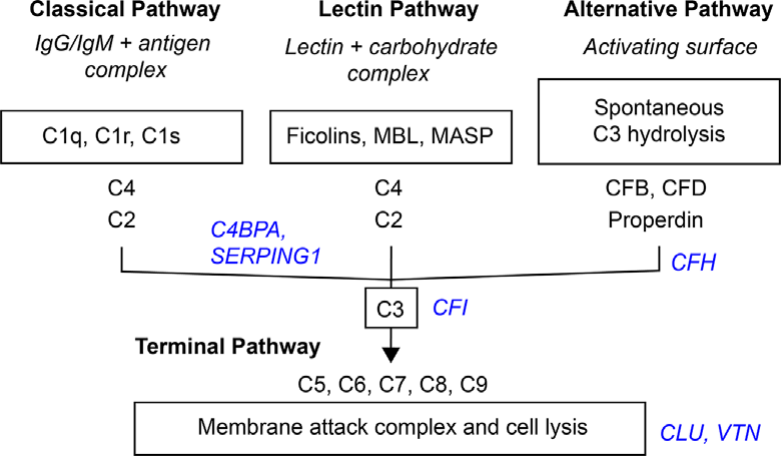
Simplified scheme of the three activation- and terminal complement pathway. Inhibitors in blue italic.

Activation of the complement system through the classical, lectin, or alternative complement pathways culminates in the common terminal pathway, which results in the formation of the membrane-lysing pore-structure termed the membrane attack complex (MAC) also known as the terminal component complex (TCC) (**Figure 6**) (54).

Although several studies have investigated circulating complement components in neonates (5), each of them have only partially characterized the complement system. Our study represents the first to characterize the development of the circulating complement system trajectories as a whole over the first week of life in two independent cohorts (**Table 2**, **Supplementary Table 9**) including whole blood transcriptomics. Neonates are relatively deficient in complement components, which increases their susceptibility to infections during the first months of life (5). The current consensus is that complement components are not able to cross the placental membrane under physiological conditions, and that all complement proteins identified in newborn blood therefore are synthesized by the fetus/newborn, and mainly by hepatocytes (55). It should be noted that C3 and C7 may be transferred from the fetus to the mother, while C1RL may be a tissue (placenta) leakage protein detected in newborn plasma (56).

**Table 2:** Change of central complement system proteins at DOL-1, 3, and 7 (0% equals no change relative to at birth).

Protein names	GN	DOL1	DOL3	DOL7	Protein level	Function	UPID
Complement C1q subcomponent B	C1QB	*−1.3%*	13.7%	**34.7%**	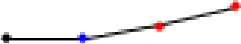	C	P02746
Complement C1q subcomponent C	C1QC	*6.2%*	18.9%	26.0%	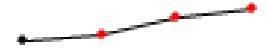	C	P02747
Complement C1r subcomponent	C1R	*12.4%*	**37.1%**	**30.6%**	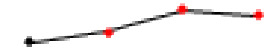	C	P00736
Complement C1s subcomponent	C1S	*4.1%*	**22.1%**	**24.1%**	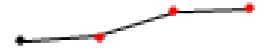	C	P09871
Complement C2	C2	*12.9%*	18.5%	*18.3%*	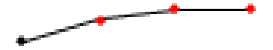	C	P06681
Complement C3	C3	*4.1%*	*2.3%*	*7.4%*	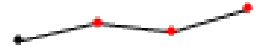	C, L, A	P01024
Complement C4-B	C4B	*6.7%*	**32.1%**	22.7%	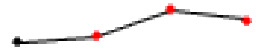	C	P0C0L5
Complement factor B	CFB	**27.4%**	**50.5%**	**63.1%**	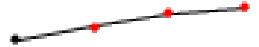	A	P00751
Complement factor D	CFD	*−16.8%*	−17.7%	−20.2%	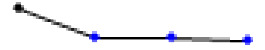	A	P00746
Properdin	CFP	*9.6%*	*−15.7%*	**−31.0%**	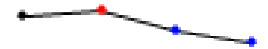	A	P27918
Prothrombin	F2	*−1.0%*	*7.8%*	*2.6%*	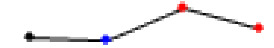	C, L, A	P00734
Ficolin-2	FCN2	*−28.7%*	*−1.5%*	*−21.5%*	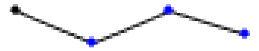	L	Q15485
Ficolin-3	FCN3	*−20.7%*	*−43.5%*	**−46.5%**	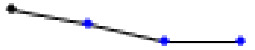	L	O75636
Complement C5	C5	*6.6%*	**26.4%**	*18.8%*	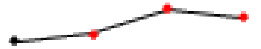	MAC	P01031
Complement component C6	C6	**69.7%**	**28.8%**	23.3%	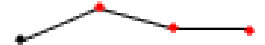	MAC	P13671
Complement component C7	C7	*3.5%*	**−12.1%**	**−28.3%**	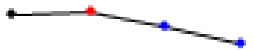	MAC	P10643
Complement component C8 alpha	C8A	*11.2%*	**46.4%**	**72.9%**	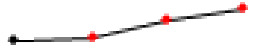	MAC	P07357
Complement component C8 beta	C8B	*22.0%*	**37.4%**	**53.6%**	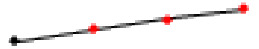	MAC	P07358
Complement component C8 gamma	C8G	**49.9%**	**113.2%**	**162.0%**	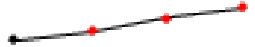	MAC	P07360
Complement component C9	C9	**75.8%**	**115.2%**	**253.2%**	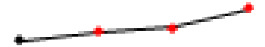	MAC	P02748
Alpha-2-macroglobulin	A2M	**−5.8%**	*−1.3%*	*0.5%*	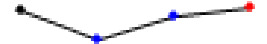	I	P01023
C4b-binding protein alpha chain	C4BPA	*1.8%*	*17.3%*	**44.8%**	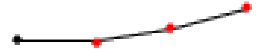	I	P04003
Complement factor H	CFH	*−4.7%*	*0.3%*	10.9%	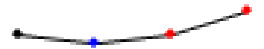	I	P08603
Complement factor I	CFI	*2.7%*	*−3.9%*	*14.1%*	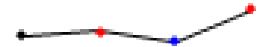	I	P05156
Clusterin	CLU	*−12.9%*	*5.8%*	**29.5%**	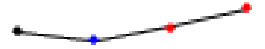	I	P10909
Carboxypeptidase B2	CPB2	*−4.0%*	*−0.5%*	*4.2%*	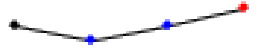	I	Q96IY4
Carboxypeptidase N catalytic chain	CPN1	*22.8%*	*0.3%*	*−1.2%*	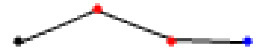	I	P15169
Vitamin K-dependent protein S	PROS1	*−6.1%*	**21.2%**	**26.4%**	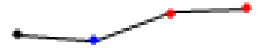	I	P07225
Plasma protease C1 inhibitor	SERPING1	*−10.2%*	*8.2%*	*−25.9%*	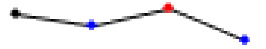	I	P05155
Vitronectin	VTN	*−5.7%*	*0.9%*	13.5%	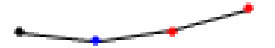	I	P04004

Gray italic: p-value > 0.05, black: p-value < 0.05, black bold: q-value < 0.05. Trend marker red (blue): positive (negative) change compared to DOL0. Pathways from Gene Ontology without further curation: C, classical; L, lectin; A, alternative; I, complement inhibitor; MAC, membrane attack complex.

### The Classical Complement Pathway Components Increase Over the First Week of Life

The classical pathway is activated, primarily on mucosal surfaces, by binding of the C1 complex (C1q, C1r, and C1s) to surface‐bound immune complexes, mainly IgG or IgM (57). C1 activation initiates the terminal pathway and the formation of the MAC transmembrane pore-structure on the target membrane.

**Figure 7:**
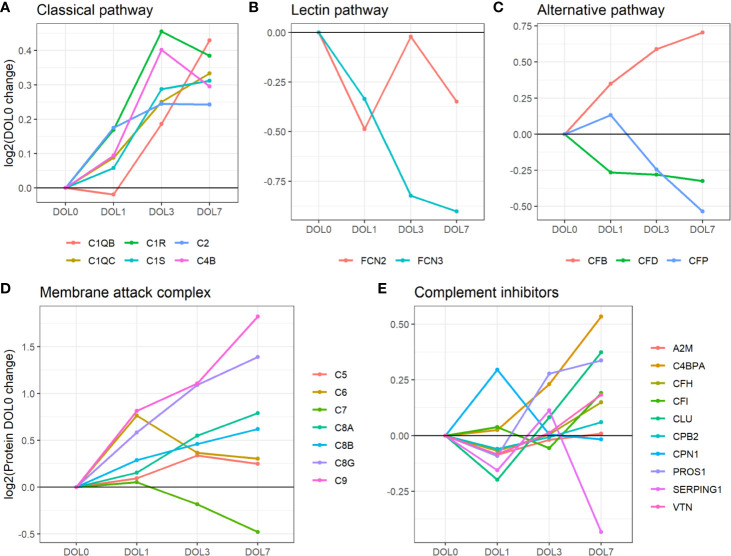
Average change of complement proteins grouped by their function across the first week of life compared to DOL0. **(A)** classical pathway, **(B)** lectin pathway, and **(C)** alternative pathway. **(D)** Membrane attack complex (MAC) proteins, **(E)** complement inhibitors. Pathway activations from Gene Ontology without further curation.

We identified C1q (chains A, B, and C), C1r, C1s, C4 (isotype B), and C2 components. While the data sufficed for the identification of C1q chain A, they did not meet our stringent requirements for robust quantification, i.e., quantitative analysis of C1qA was omitted. Plasma concentrations of the C1 complex (C1qB, C1qC, C1r, and C1s), C4B, and C2 from the classical component pathway increased over the first week of life (**Figure 7A**, **Table 2**). C1r and C1s were significantly increased on DOL3, and C1qB on DOL7 compared to DOL0. Highly similar changes were seen in the validation cohort (**Supplementary Tables 8**, **9**).

While the trajectories of C1q chain B and C demonstrated a similar increase, only C1qB was significantly increased at DOL7 with C1qC at near significance levels (p-value 0.026, q-value 0.060). C1q is predominantly synthesized by blood-cells, whereas C1r and C1s, like most complement components, are synthesized mainly by hepatocytes (58). The difference in synthesis location likely explains the slightly different abundance pattern of C1q lacking a significant increase on DOL3. The increased concentration of C1q is in good agreement with previous reports (5). Our study is the first to report the increased concentration of the individual B and C subunits.

Our findings demonstrate the increased levels of the components activating the classical complement pathway, which is supported by the finding of increasing levels of C4B rather than C4A. In humans, C4 exists in isotypes A and B, and following activation of the C1 complex, both isotypes are cleaved into alpha and beta-chains (59). C4A beta forms an amide bond which has good efficiency in binding amino group-rich immune complexes whereas C4B beta forms an ester bond which has good efficiency in binding carbohydrate-rich cellular surfaces (60). This makes C4B the more functionally active isotype to activate C3 through the classical- and lectin pathway (61). As a result, a deficiency of C4B increases vulnerability to bacterial and viral infections, whereas C4A deficiency is linked to autoimmune diseases (61).

Overall, the average plasma concentration of classical pathway components was significantly increased over the first week of life, and as early as 24 h after birth.

### The Lectin Complement Pathway Components Decrease Over the First Week of Life

In contrast to the increase in components of the classical complement pathway, plasma concentrations of complement components involved in initiating the lectin complement pathway decrease over the first week of life. The lectin pathway is initiated by binding of mannose-binding lectin (MBL), collectin 11, and ficolins (FCN1, FCN2, and FCN3) to oligosaccharides and acetylated residues on the surface of bacteria, viruses, and dying cells. Following binding, mannan-binding lectin serine protease 1 (MASP-1) or 2 is recruited into the MBL complex, which similarly to the classical complement pathway results in cleavage of C2 and C4 and formation of the C4bC2b complex. MASP-1 is required for activation of MASP-2, and of the two only MASP-2 can cleave C4 (62). The resulting C4bC2b complex triggers the enzymatic cleavage of C3 into C3a and C3b, and initiates the terminal complement pathway (62).

Of the six lectin complement pathway pattern-recognition proteins, we identified three in our plasma proteomics dataset: FCN-2, FCN-3, and MASP1. While the data sufficed for the identification of MASP1, they did not meet our stringent requirements for robust quantification, i.e., quantitative analysis of MASP1 was omitted. FCN-3 showed a downward trend during the first week of life and was significantly reduced at DOL7 (**Figure 7B**) in the main cohort, whereas the reduced levels at DOL7 were not statistically significant in the validation cohort (**Supplementary Table 10**). Since neither of the two underlying genes are transcribed/translated in blood cells, no corresponding mRNA information was obtained (63). The lack of feasibility to detect and/or solidly quantify lectin complement pathway proteins, might be explained by a presumed low plasma concentration, and warrants targeted follow-up studies.

Serum concentrations of lectin pathway factors FCN-1, FCN-2, FCN-3, MASP-2 (64), and MBL (65, 66) are reportedly low in newborns compared to infants, children, and adults (67). Ficolins are reportedly lower in neonates than in older children (68). This is the first study to report on the FCN-3 concentration trajectory during the first week of life. The measured decreased plasma concentrations of FCN-3 through the first week of life would be expected to further be associated with impaired activation of the lectin complement pathway in early life, and indicates that the increase of lectin pathway component levels takes place after the first week of life.

Overall, our findings indicate that the levels of activating components in the lectin complement pathway remain relatively deficient in the first week of life, a finding supported by negligible protein synthesis.

### The Alternative Complement Pathway Components Display a Binomial Distribution

Compared to classical- and lectin complement pathways, changes in components of the alternative complement pathway displayed a binomial distribution. Our plasma proteomic workflow provided us with identification and quantification of three proteins associated with activation of the alternative complement pathway (69) (**Figure 7C**). We measured a statistically significant decrease of properdin (aka complement factor P/CFP) at DOL7, and an overall decreasing trend during the first week of life, whereas complement factor B (CFB) continuously increased from DOL1 onward to DOL7. A similar protein trajectory was found in the validation cohort for CFB, whereas the levels of CFP did not achieve statistical significance (**Supplementary Table 9**).

CFB and CFP are essential for activation of the alternative complement pathway, and newborns have reduced plasma concentration compared to adults (5). Adult levels of CFB are reportedly achieved within 6 months after birth, in contrast to CFP-levels that remain low at 6 months of age (5). Here we demonstrated for the first time that these trajectories are established as early as 24 h after birth. CFP is the only complement regulator that enhances complement activation, by stabilizing the C3bBb-complex. It is mainly synthesized outside the liver by neutrophils, monocytes, and primary T cells, and can be secreted upon stimulation (70, 71). The CFP mRNA levels did not show any significant change during the first week of life (fold-change < 0.2, p-value > 0.05) (7), indicating insufficient CFP synthesis to maintain the DOL0-plasma levels, a lack of compensatory protein synthesis, and a presumed absence of leukocyte stimulation.

Overall, our findings indicate that the levels of activating components in the alternative complement pathway remain low with negligible synthesis in the first week of life.

### The Terminal Complement Pathway Components Increase Over the First Week of Life

The MAC is the terminal point of the terminal complement pathway and complement activation, and the transmembrane pore-structure which causes cell lysis and ultimately cell death (54).

Our plasma platform allowed us, for the first time, to quantify all major MAC components, C5-9 (**Figure 7D**). At DOL3 and DOL7, all but one of the major components of MAC showed significant abundance increases (C5, C6, C8, and C9). The notable exception was C7, which showed a significant decrease in plasma levels during the first week of life. Again, similar protein trajectories were found in the validation cohort (**Supplementary Table 9**).

The MAC components are mainly synthesized by hepatocytes. In this regard, C7 is unique in being synthesized by blood cells in addition to hepatocytes (72–74). Newborns demonstrate near-adult levels of C7, as opposed to low levels of the remainder of MAC components (5, 75). C7 is often the rate-limiting step in MAC-formation and a decrease of C7 would potentially inhibit MAC formation, while local C7 synthesis at the site of inflammation might enhance MAC formation (72). The decrease of C7 from DOL3 onward is also noteworthy as we were unable to even detect the corresponding mRNA in whole blood.

Low levels of the MAC components at baseline followed by their increase, apart from C7, could indicate MAC maturation to support innate immunity.

### The Complement Inhibitors

Inhibitors are central in controlling complement system activity in order to limit damage to the site of inflammation and protect healthy host-cells.

Out of the 10 complement system inhibitors identified in our analysis, A2M was reduced on DOL1. From DOL1 onward, five showed a subsequent increase (**Figure 7E**) based on adjusted (CLU, vitamin K-dependent protein S/PROS1, and C4b-binding protein alpha-chain/C4BPA) or unadjusted (CFH and vitronectin/VTN) p-values at DOL7 compared to DOL0. A similar trajectory of PROS1, C4BPA, and CFH was seen in the validation cohort (**Supplementary Table 9**).

The general decrease of complement inhibitors 24 h after birth supports our finding of an acute phase response on DOL1, driven by an increase of acute phase proteins and complement proteins, and the decrease of several complement system inhibitors. A2M is a protease inhibitor and it has been reported that A2M binds covalently to and inhibits MASP (73), although the inhibitory functions of A2M on the complement system are still debated (74). In our study the decreased A2M-levels at DOL1 returned to baseline levels at DOL3. This is the first study to report on the A2M levels in the first week of life, and reportedly infants and children have increased A2M-levels compared to adults, indicating that the increased trajectory of A2M likely continues past DOL7 (42).

CLU and VTN inhibit formation of the MAC (76), and newborns have lower levels when compared to adults (42, 77). The increased MAC inhibitor and the increasing abundances of the MAC components therefore clearly indicate a readying of the MAC for the postnatal period during the first week of life.

Newborns have low levels of CFH compared to adults (5), and the steady levels of CFH and sudden increase at DOL7 could indicate the early preparation for the alternative complement pathway, in line with the detected increase of CFB at DOL7 (**Supplementary Table 9**).

The function of PROS1 in the complement system remains incompletely understood. However, PROS1 has been suggested to redirect C4BP to the surface of apoptotic cells to control activation of the classical complement pathway. PROS1 has high affinity for negatively charged phospholipid phosphatidylserine, which is expressed and exposed on cells in the initial stage of apoptosis. In human plasma, 60–70% of PROS1 circulates bound in a complex with C4B. It is reported that PROS1 mediates binding of C4BP to apoptotic cells, thereby controlling complement activation (78). Newborn levels of PROS1 and C4BP are lower than adult levels (78). PROS1 also has anticoagulant effects lost upon binding to C4BP. The similar increase of PROS1 and C4BP makes it possible that the increased level of PROS1 is related to regulating the increase of complement system components. However, further studies are required.

Altogether, our findings demonstrated the consistent and robust increase of complement components in the classical activation pathway over the first week of life, while components of the lectin- and alternative pathway lacked a clear developmental trajectory. Additionally, all components of the MAC except the rate-limiting C7 were increased at DOL7. The absence of blood cell expression of C7, SAA1, and CFP indicates that blood cell stimulation is not a main initiator of the protein synthesis in the first week of life, and that the maturation of the complement components is therefore mainly driven by ontogeny rather than bacterial stimulation. Finally, we measured a decrease of most complement system inhibitors at DOL1 and subsequent recovery and increase at DOL7. Newborns are deficient in most complement components and the increasing plasma-levels indicate that the complement system components are approaching adult levels and maturation. However, future studies should be performed to investigate if the complement system activity follows the measured concentrations of complement system components. Speculatively, the apparent prioritization of synthesis of components related to the classical and terminal complement pathway, could be a means of obtaining a first complete complement response to pathogens as quickly as possible during a resource constrained immune system development.

### Whole Blood Messenger RNA Levels of the Complement System Poorly Reflect Protein Plasma Levels

The inconsistency between protein and mRNA levels for several complement components, led us to perform a correlation analysis for the complement pathway data. We extracted all complement proteins for which we had corresponding whole blood mRNA information.

**Figure 8:**
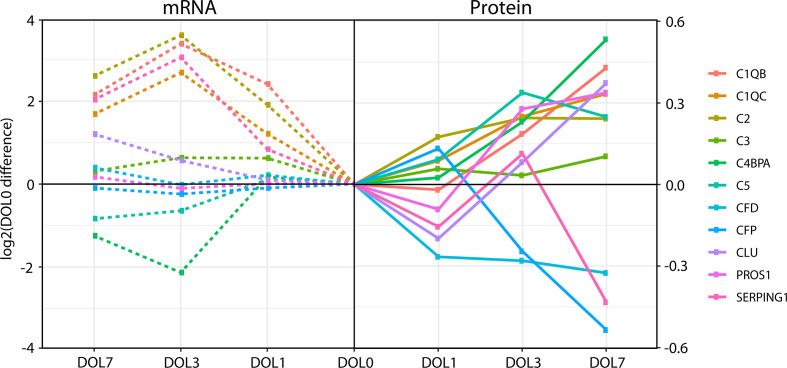
Divergence of complement plasma protein and whole blood messenger RNA (mRNA) levels, as compared to DOL0, of the 11 complement proteins with detected whole blood mRNA.

While the majority of the complement proteins with corresponding whole blood transcripts increased with age (**Figure 8**), the corresponding mRNA levels had a binominal distribution, with one group increasing from DOL0 to DOL3 and subsequent leveling off at DOL7, while the other group showing little abundance differences during the first week of life (**Figure 8**). Accordingly, there was a weak correlation between the change in protein plasma levels and change in whole blood mRNA levels (all pairs R < 0.3, p-value > 0.05). Of note, when we performed the correlation analysis using DOL average rather than individual participant data, we achieved much higher (R −0.67 to 0.88) yet still statistically insignificant (p-value > 0.05) correlation coefficients. This observation suggests large protein-to-RNA level variations between participants, or could represent concordance of mRNA and protein level data for only a small subset of complement protein. Indeed, of the 30 monitored complement proteins, the mRNA levels reflected the protein level changes for the following seven complement proteins (average R > 0.3): C1QB (R = 0.88), C1QC (R = 0.86), C2 (R = 0.90), C3 (R = 0.85), CLU (R = 0.74), SERPING1 (R = 0.79), and complement factor D/CFD (R = 0.49).

Differences between the mRNA/transcriptomics and proteins/proteomics information highlight the fact that there is no transcriptomics equivalent to the plasma proteome. The plasma proteome is a systemic body fluid integrating protein reflecting dynamic processes of protein expression and degradation across different organ systems and body compartments, while the whole blood transcriptome provides transcriptomic information from whole white blood cell fraction only (non-nucleated red blood cells having proteins but no transcripts), with neutrophils having lower transcript abundance making a more minor contribution. As the white blood cells are removed during the plasma preparation, whole blood transcriptomics and plasma proteomics map the two complementary, mutually exclusive blood fractions. Indeed, integration of the transcriptome and proteome is key to elucidate meaningful physiologic changes and generate mechanistic hypotheses to study the immune ontogeny of the newborn.

### Increased Immunoglobulin M and J Chain Synthesis

The increased levels of components related to the classical complement activation pathway prompted us to investigate IgM and IgG, the main target molecules for the C1-complex and thereby activators of the classical complement pathway. IgM cannot cross the placental barrier and is the first antibody synthesized by peripheral blood lymphocytes during primary infections, thereby highlighting its central role in the initial immune response (79, 80). IgM is a pentamer connected with joining chains (J chain), which regulates the polymerization of the molecule for efficient secretion (79, 81).

**Figure 9:**
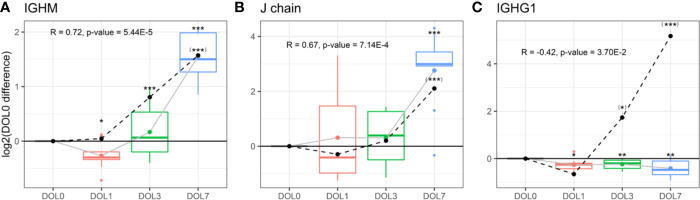
Protein and messenger RNA (mRNA) levels (dotted line) of **(A)** IgM, **(B)** J chain, **(C)** IgG1 across the first week of life compared to DOL0, with mean abundance difference indicated by dots connected by lines. Protein to RNA correlation and p-value given. Statistics compared to DOL0: q-value protein (mRNA) <0.05: *(*), <0.01: **(**), <0.001: ***(***).

Plasma levels of IgM increased over the first week of life (**Figure 9A**), a finding confirmed in the validation cohort (**Supplementary Figures 6A, B**). J chain levels also increased, mirroring the IgM concentration trajectories (**Figure 9B**), with a strong positive Pearson’s correlation that was statistically significant (R = 0.73, p-value = 2.44E−5). In accordance with plasma concentrations, we measured similar increased mRNA level trajectories for the IgM and J chain when compared to the plasma proteins and found a significant positive correlation between plasma and mRNA levels (**Figures 9A, B** dotted line).

The physiologic average serum level of IgM is reportedly 18.5 mg/dl (15% of adult levels) in the first month of life, and this increases 3-fold in months 1–5 of life reaching adult levels by 1–2 years of age (82). The present study demonstrated that IgM levels increased from DOL3 onward in the first week of life, and based on the data an incant at DOL7 can be expected to have IgM concentrations of 38 to 54 mg/dl, when assuming average IgM concentrations at DOL0. J chain is required for Ig-transport across the mucosal epithelium, e.g., for secretion. Whether J chain regulation occurs at the protein level remains to be determined, and there are conflicting views on the origins of some of the J chain secreting cells (81). Based on our findings, there was J chain expression in whole blood and there were ontogeny-induced increases at both the cell transcriptional and plasma-protein levels.

### Insufficient Immunoglobulin G Synthesis

IgG antibodies are the most abundant Ig-class in the circulation, especially in the fetus through placental transfer mediated by the FcRn receptors (82). Placentally transferred IgG provide protection in the first weeks of life due to the relatively long *in vivo* half-life of IgG (~3 weeks) (83). Of the four IgG subclasses, IgG1 can primarily be induced by soluble protein antigens and membrane proteins, IgG2 by bacterial capsular polysaccharides, IgG3 by viral antigens (like IgG1) and induce pro-inflammatory effects, and IgG4 by repeated exposure to a non-infectious antigen (84).

**Figure 10:**
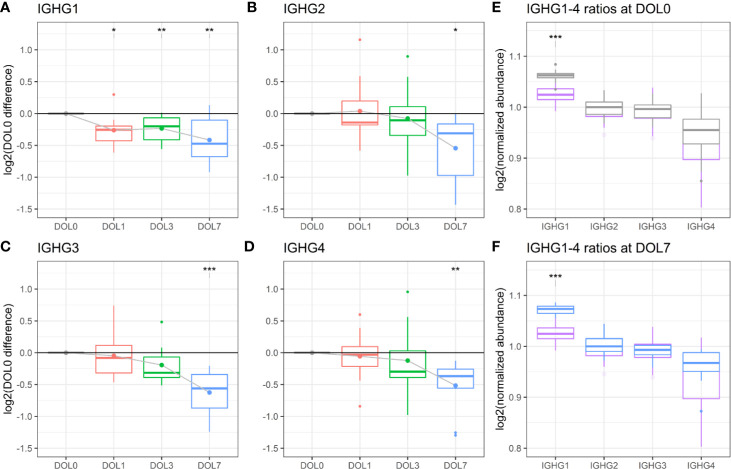
Protein levels of **(A–D)** IgG1-4 across the first week of life as compared to DOL0, with mean abundance difference indicated by dots connected by solid line. **(E)** Comparison of the ratios of IgG1-4 normalized to IgG2 between adult-levels (purple) and newborn-levels at DOL0 (gray), or **(F)** DOL7 (blue). Statistics A–D **(E, F)**: q-value <0.05: *, <0.01: **, <0.001: ***.

We measured significantly decreasing plasma concentrations of all four IgG subclasses over the first week of life, and IgG1 was significantly decreasing as early as 24 h after birth (**Figures 9C**, **10A–D**). A similar trajectory of IgG plasma-levels was seen in the validation cohort (**Supplementary Figures 7A–D**).

Serum levels of IgG1-4 are reportedly decreased in 1–5 month old infants compared to 0–30 day old newborns (82). Our study adds to the existing literature by providing granularity in the IgG1-4 plasma level trajectory over the first week of life. The measured decreases in IgG levels from birth to 5 months of age are consistent with a drop of maternal IgG levels in the newborn circulation, on top of low endogenous production. This physiologic transient hypogammaglobulinemia is a well-described phenomenon and is typically not clinically significant. Adult concentrations of most IgG-subclasses are eventually reached by 4–6 years of age (82).

We were interested in determining the timing of initiation for endogenous IgG synthesis from newborn plasma cells. We thus measured mRNA levels of IgG subclasses from DOL0, and found IGHG1 mRNA transcripts in 26 of the 30 newborns at DOL0 (validation cohort 13 of 19 newborns), indicative of endogenous IgG1 synthesis as early as DOL0. Additionally, we observed that IGHG1 mRNA levels were significantly increased on DOL3 and DOL7 (**Figure 9C**), while no mRNA was detected for IGHG2-4 in either cohort. The protein levels and mRNA levels of IGHG1 were negatively correlated (R = −0.42, p-value = 0.037), indicating that despite endogenous IgG1 synthesis as early as DOL0, plasma levels decreased over the first week of life, likely because of dominance of the *in vivo* degradation process. These findings were reproduced in the validation cohort (**Supplementary Figure 6C**).

To investigate the impact of the IgG1 synthesis on the plasma concentration, we calculated the apparent IgG1 plasma concentration half-time in our dataset to 21.1 days. This is comparable to the reported IgG1 *in vivo* half-life of 21–25.8 days (85, 86). The decline in IgG1 plasma concentration in newborns is thereby explained by the reported IgG1 *in vivo* half-life (with no synthesis). This demonstrates that IgG1 synthesis remains insignificant in the first week of life.

We performed an analysis of the inter IgG-subclass ratios. As different proteins were compared, we estimated the IgG-subclass abundances using intensity-based absolute quantitation (iBAQ) values. To enable a comparison to adult levels, we included data from a previously published proteomics study of plasma from 30 healthy adult Danes (10). To compensate for the different experimental designs, we normalized the IgG ratios to IgG2 in each dataset. Of note, normalization to IgG3 yielded similar results.

The analysis revealed that IgG1 was the most abundant IgG throughout the first week of life followed by IgG2, IgG3, and IgG4 (**Figures 10E, F**), in agreement with published literature from newborns within the first 30 days of life (82).

Of the four IgG subclasses, there is a preferential placental transport of IgG1, followed by IgG4, and to a lesser extend of IgG3 and IgG2, based on FcRn receptor affinity (55, 87). Healthy term newborns are born with similar to increased IgG-concentrations compared to the mother (88). Because we did not have access to the maternal IgG-levels, we obtained a previously published proteomics dataset of plasma from 30 healthy adult Danes (**Figures 10E, F**). Newborns had a significantly increased IgG1 to IgG2-4 ratio, when compared to adults. The findings were reproduced in the validation cohort (**Supplementary Figures 7E, F**).

IgM, IgG1, and IgG3 antibodies are efficient activators of the complement system through the classical pathway, whereas a high epitope density is needed of IgG2, and IgG4 is ineffective (89, 90). The relatively elevated concentration of IgG1 in the newborns compared to adults, combined with the specific synthesis of this subclass, and the increase in IgM, suggest a central role of the classical pathway of complement activation for newborn immunity.

## Conclusion

Our study presented a mapping of the plasma proteome across the first week of human life in a paired African cohort with an independent geographically distinct validation cohort in Australasia. Newborns are primarily dependent on innate immunity to shield them from infection, and we characterized the ontogeny of several innate immune components over the first week of life. Specifically, we found increased plasma and mRNA levels of all major components related to the classical and terminal complement pathways, including the C1 complex and the target molecule IgM, as early as 24 h after birth. All components of the MAC, except C7 which is at near-adult levels at birth, increased by DOL7, as well as most complement system inhibitors, suggesting a central role for the classical complement pathway for neonatal immunitysuggesting a central role for the classical complement pathway for neonatal immunity (91–93). With respect to complement components, we noted significant differences between plasma protein concentrations and whole blood mRNA counts, highlighting the value of multi-OMICS integration to gain biologic insights and generate well-informed hypotheses.

We were able to provide granularity around the trajectory of different immunoglobulin subtypes during the first week of life. We detected endogenous synthesis of the IgG1 isotype, but not IgG2-4 and the ratio of IgG1 to IgG2-4 was elevated compared to adults, highlighting the importance of IgG1 for early immunity. Levels of the maternally transferred IgG1-4 declined over the first week of life. We also detected an acute phase response accompanied by a surge in levels of acute phase proteins on the first day of life and an overall reduction of inhibitors. The functional consequences of the differences between the timepoints could not be assessed *in vitro* due to sample size restrictions, and should be investigated in further studies. Additionally, studies involving a higher number of samples, pairing with maternal samples, and higher time resolutions are needed to identify the drivers of this response.

The main strength of our study is the performance of deep proteomics analysis with small volumes of blood plasma. Even though most of our findings are not surprising, this study’s main contribution to the literature is a new conceptual framework of neonatal immune ontogeny based on a consistent developmental trajectory of the newborn’s immune system over the first week of human life across continents, as evidenced by highly similar trajectories in two geographically distinct newborn cohorts. This study is also proof of concept that high quality, reproducible systems biology research is feasible even in low resource settings.

In summary, our findings have shed additional light on functionally distinct immunobiological processes occurring in the human neonate during the first week of life and can serve as a crucial overview and hypothesis-generating resource for researchers in the field of immune ontogeny. Interpretation of our observations in the context of perinatal physiology will be key to understanding the delicately balanced regulation of the neonatal immune response.

## Data Availability Statement

The datasets presented in this study can be found in online repositories. The names of the repository/repositories and accession number(s) can be found in the article/**Supplementary Material**.

## Ethics Statement

The studies involving human participants were reviewed and approved by Local Ethics Committee-approved protocol (MRC SCC 1436 and IMR IRB#1515 and MRAC #16.14). Written informed consent to participate in this study was provided by the participants’ legal guardian/next of kin.

## The EPIC Consortium in Alphabetical Order

Nelly Amenyogbe, Asimenia Angelidou, Tue Bjerg Bennike, Anita H.J. van den Biggelaar, Cai Bing, Ryan R. Brinkman, Kim-Anh Lê Cao, Momoudou Cox, Alansana Darboe, Joann Diray-Arce, Reza Falsafi, Benoit Fatou, Davide Ferrari, Rebecca Ford, Erin E. Gill, Robert E.W. Hancock, Daniel J. Harbeson, Simon D. van Haren, Daniel He, Samuel J. Hinshaw, Olubukola T. Idoko, Beate Kampmann, Ken Kraft, Wendy Kirarock, Tobias R. Kollmann, Amy H. Lee, Ofer Levy, Mehrnoush Malek, Geraldine Masiria, John Paul Matlam, Jorjoh Ndure, Jainaba Njie-Jobe, Oludare Olumade, Rym Ben-Othman, Al Ozonoff, Matthew A. Pettengill, William S. Pomat, Peter C. Richmond, Elishia Roberts, Gerard Saleu, Guzmán Sanchez-Schmitz, Casey P. Shannon, Amrit Singh, Kinga K. Smolen, Hanno Steen, Scott J. Tebbutt, Diana Vo.

## Author Contributions

OL, TK, HS, RH, ST, AO, AB, and BK were the overall project and core leads. OI and BK established the main cohort. WP and AB established the validation cohort. JD-A, SH, RB-O, and KS processed the samples. TB and BF generated the proteomics data. TB analyzed the proteomics data. RFa, RFo, EG, AL, and RH generated and analyzed the RNA seq data. TB, BF, and HS interpreted the results. TB wrote the manuscript draft with BF and HS. Substantial help was received from the entire EPIC-HIPC. All authors contributed to the article and approved the submitted version.

## Funding

TK’s laboratory is supported by a Michael Smith Foundation for Health Research Career Investigator Award. OL’s laboratory is supported by the following U.S. NIH/NIAID awards: Molecular Mechanisms of Combination Adjuvants (1U01AI124284-01), Adjuvant Discovery Program Contract No. HHSN272201400052C and Human Immunology Project Consortium (U19AI118608) as well as an internal Boston Children’s Hospital award to the *Precision Vaccines Program*. BK is supported by grants from the MRC/UKRI (MC_UP_A900/1122, MC_UP_A900/115, MR/R005990/1), and the additional field team and laboratory staff at the MRC Unit in The Gambia. Recruitment of the cohort of newborns in Papua New Guinea was funded by seed funding awarded to AB from the Wesfarmers Centre of Vaccines and Infectious Diseases, Telethon Kids Institute. The work in RH’s lab was initially supported by the Canadian Institutes for Health Research grant #FDN-154287 and he holds a Canada Research Chair in Health and Genomics and a UBC Killam Professorship. RRB’s laboratory is supported by an award from Natural Sciences and Engineering Research Council of Canada. The Lundbeck Foundation (R181-2014-3372), The Carlsberg Foundation (CF14-0561), and A.P. Møller Foundation are acknowledged for grants enabling TB’s work. K-ALC is supported in part by the National Health and Medical Research Council (NHMRC) Career Development fellowship (GNT1087415).

## Conflict of Interest

OL is a named inventor on patents regarding bactericidal/permeability increasing protein (BPI), including “Therapeutic uses of BPI protein products in BPI-deficient humans” (WO2000059531A3) and “BPI and its congeners as radiation mitigators and radiation protectors” (WO2012138839A1). RRB has ownership interest in Cytapex Bioinformatics Inc.

The remaining authors declare that the research was conducted in the absence of any commercial or financial relationships that could be construed as a potential conflict of interest.
