# Supplementary material for: Preparing for Life: Plasma Proteome Changes and Immune System Development During the First Week of Human Life
Source: Front Immunol. 2020 Oct 20;11:578505. doi: 10.3389/fimmu.2020.578505 (PMC7732455; doi:10.3389/fimmu.2020.578505)
Supplement: Supplementary file 1 [file DataSheet_1.pdf]

Supplementary Material

1 Supplementary Data

All data are publicly available. The mass spectrometry RAW data and search results have been deposited to the ProteomeXchange consortium via the PRIDE partner repository, and is available with the data set identifier PXD019817 as well as archived on ImmPort (https://immport.niaid.nih.gov/home) under accession numbers SDY1256 and SDY1412. The transcriptomics data have been submitted to the NCBI Gene Expression Omnibus under accession numbers GSE111404 and GSE123070.

2 Supplementary Figures and Tables

2.1 Supplementary Figures

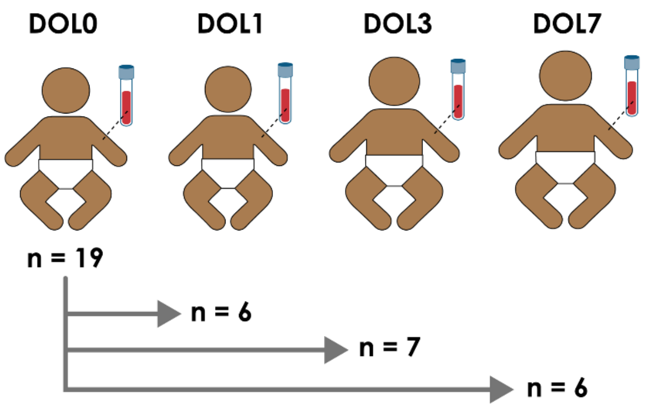

Supplementary Figure 1. Study design and number of enrolled newborns in the validation cohort from Papua New Guinea (PNG), Australasia.

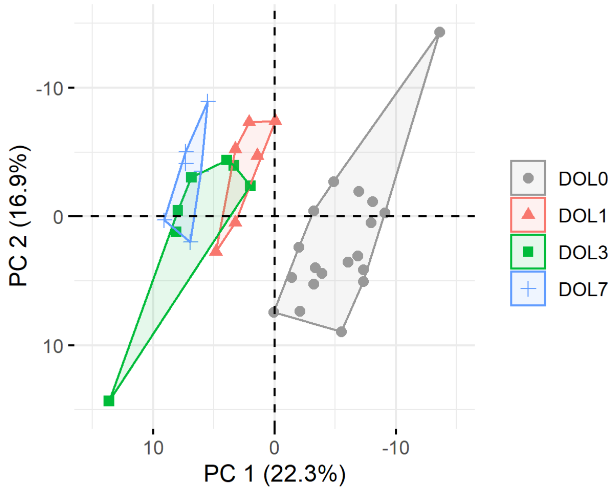

Supplementary Figure 2. Unsupervised principal component analysis (PCA) plot of all quantifiable proteins in the validation cohort, demonstrates that the largest variance in the dataset, principal component 1 (PC1), separates samples based on DOL, similarly to the main cohort. Explained variance given as percentages.

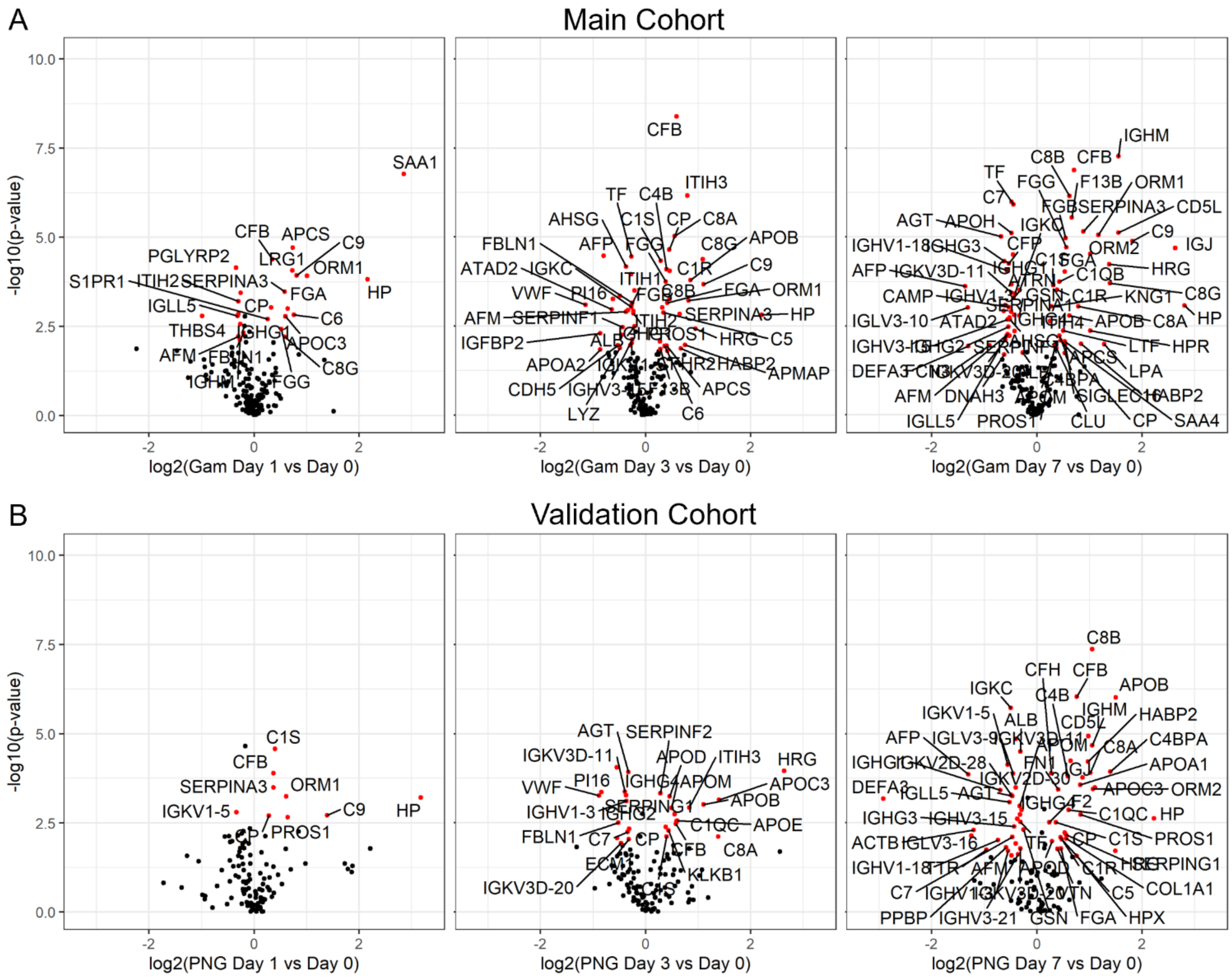

Supplementary Figure 3. Volcano plots of all quantifiable proteins in A) the main and B) validation cohort. Significantly regulated proteins (q-value < 0.05, absolute log2[fold change vs DOL0] > 0.2) are marked with red and given gene names when available.

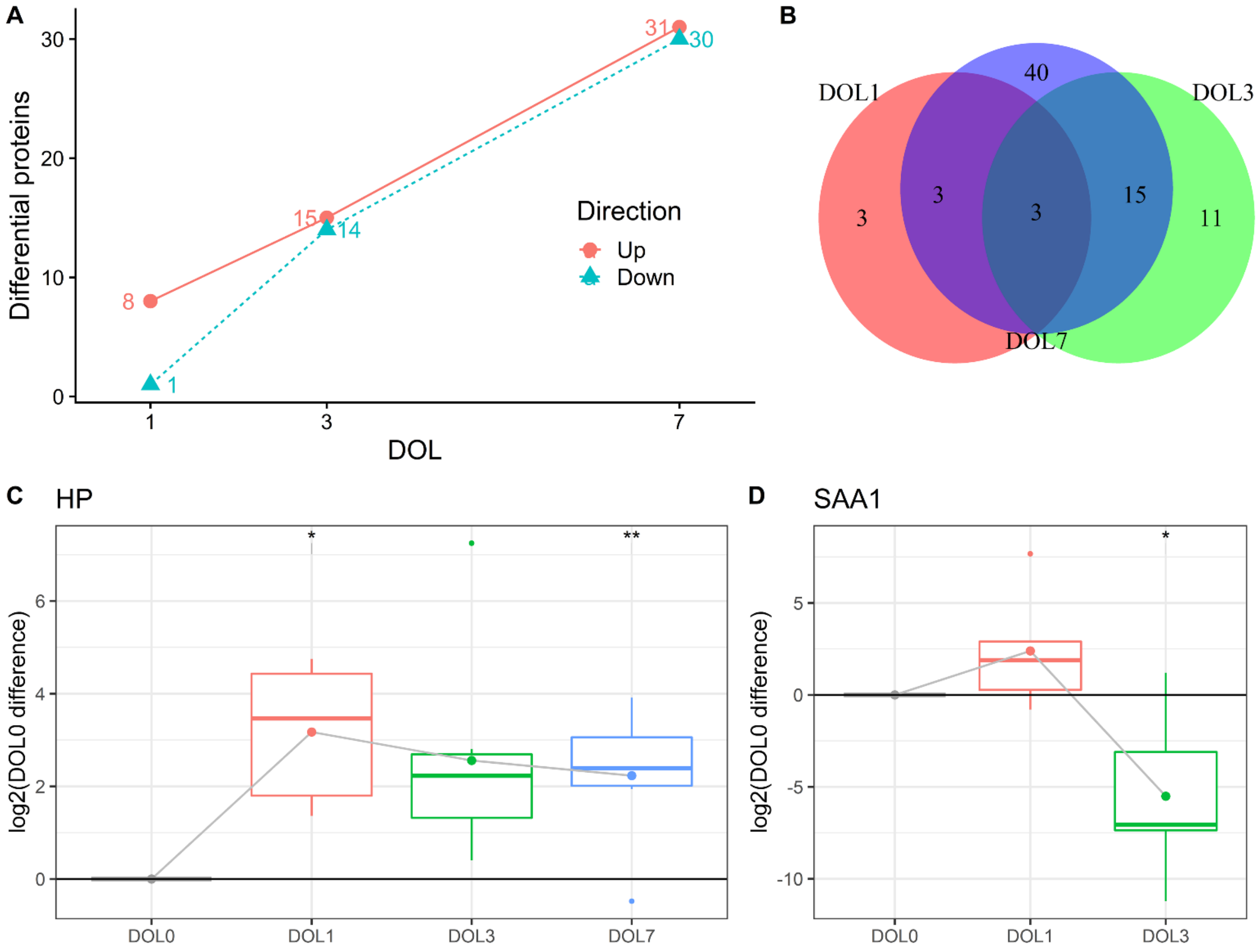

Supplementary Figure 4. Validation cohort. Differentially regulated plasma proteins (q-value < 0.05) compared to day of life (DOL) 0. A) We identified a robust trajectory of differentially expressed proteins over the first week of life. B) Overlap of regulated proteins. Protein

regulations of C) Haptoglobin (HP), D) Serum amyloid A1 (SAA1) normalized to DOL0, with mean abundance difference indicated by dots and solid line. q-value * < 0.05, **: < 0.01, ***: < 0.001..

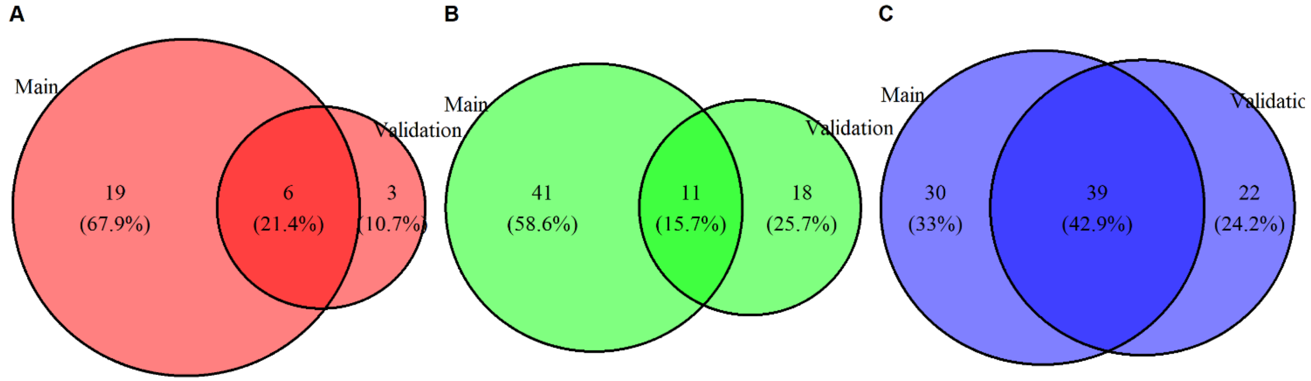

Supplementary Figure 5. Overlap in significantly differentiated proteins in the main and validation cohorts on A) DOL1, B) DOL3, and C) DOL7, compared to DOL0.

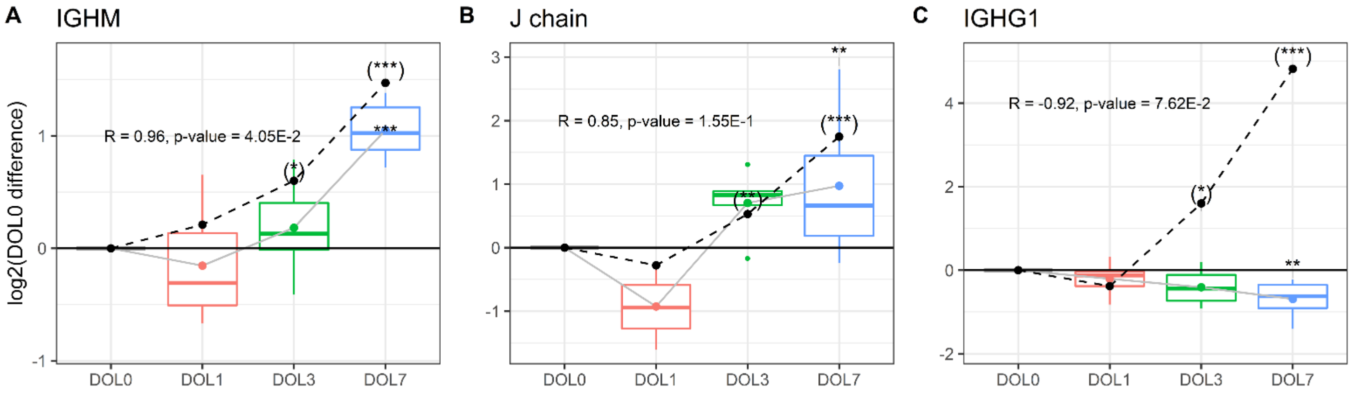

Supplementary Figure 6. Validation cohort. Protein and mRNA levels (dotted line) of A) IgM, B) J chain, C) IgG1 across the first week of life compared to DOL0, with mean abundance difference indicated by dots and solid line. Protein to RNA correlation and p-value given. Statistics compared to DOL0: q-value protein (or mRNA) < 0.05: *(*), < 0.01: ** (**), < 0.001: *** (***).

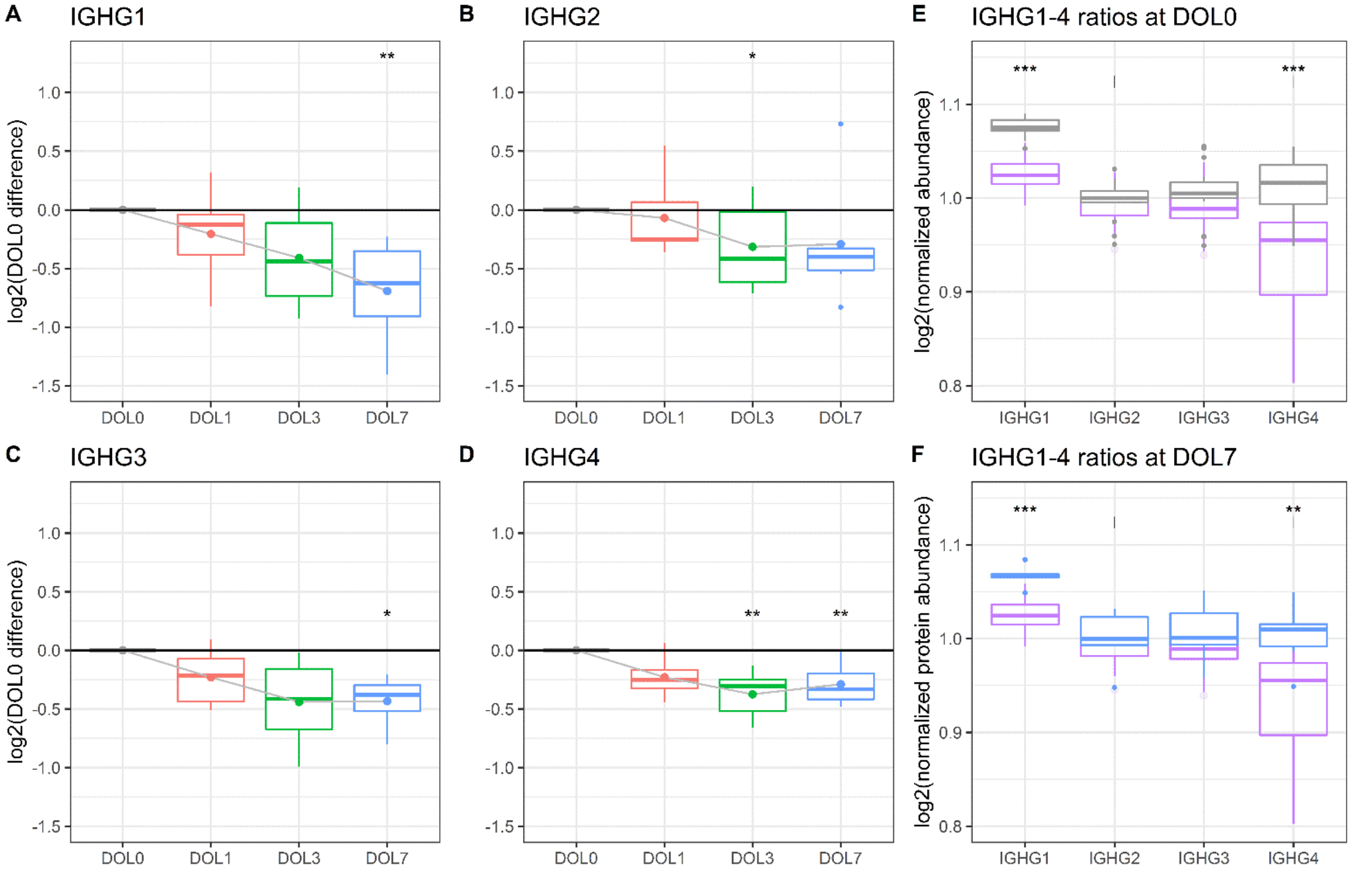

Supplementary Figure 7. Validation cohort. Protein levels of A-D) IgG1-4 across the first week of life as compared to DOL0, with mean abundance difference indicated by dots and solid line. E) Comparison of the ratios of IgG1-4 normalized to IgG2 between adult-levels (purple) and newborn-levels at DOL0 (grey), or F) DOL7 (blue). Statistics A-D (E, F): q-value < 0.05: *, < 0.01: **, < 0.001: ***.

2.2 Supplementary Tables

FC log2	Protein	Gene Name	UPID	p-value	q-value
2.85	Serum amyloid A-1 protein	SAA1	P0DJI8	1.68E-07	3.00E-05
2.16	Haptoglobin	HP	P00738	1.48E-04	3.30E-03
1.01	Alpha-1-acid glycoprotein 1	ORM1	P02763	1.21E-04	3.07E-03
0.81	Complement component C9	C9	P02748	1.18E-04	3.07E-03
0.76	Complement component C6	C6	P13671	1.48E-03	1.54E-02
0.74	Serum amyloid P-component	APCS	P02743	1.94E-05	1.73E-03
0.73	Leucine-rich alpha-2-glycoprotein	LRG1	P02750	8.38E-05	2.99E-03
0.64	Fibrinogen alpha chain	FGA	P02671	9.88E-04	1.35E-02
0.59	Apolipoprotein C-III	APOC3	P02656	1.65E-03	1.54E-02
0.58	Complement component C8 gamma chain	C8G	P07360	6.22E-03	3.95E-02
0.58	Alpha-1-antichymotrypsin	SERPINA3	P01011	3.30E-04	6.33E-03
0.52	Fibrinogen gamma chain	FGG	P02679	3.73E-03	2.89E-02
0.35	Complement factor B	CFB	P00751	4.16E-05	2.47E-03
0.33	Ceruloplasmin	CP	P00450	9.27E-04	1.35E-02
0.26	Sphingosine 1-phosphate receptor 1	S1PR1	P21453	1.97E-03	1.75E-02
-0.20	Fibulin-1	FBLN1	P23142	4.84E-03	3.44E-02
-0.26	Immunoglobulin kappa variable 3-15	IGKV3-15	P01624	3.56E-04	6.33E-03
-0.26	Ig gamma-1 chain C region	IGHG1	P01857	2.68E-03	2.27E-02
-0.27	Ig mu chain C region	IGHM	P01871	7.44E-03	4.57E-02
-0.30	Inter-alpha-trypsin inhibitor heavy chain H2	ITIH2	P19823	6.27E-04	1.01E-02
-0.30	Immunoglobulin lambda variable 2-8	IGLV2-8	P01709	1.42E-03	1.54E-02
-0.30	Afamin	AFM	P43652	6.06E-03	3.95E-02
-0.31	Immunoglobulin lambda-like polypeptide 5	IGLL5	B9A064	1.60E-03	1.54E-02
-0.34	N-acetylmuramoyl-L-alanine amidase	PGLYRP2	Q96PD5	7.08E-05	2.99E-03
-0.99	Thrombospondin-4	THBS4	P35443	1.61E-03	1.54E-02

Supplementary Table 1. Significant proteins in the main cohort DOL1 vs DOL0 (q-value < 0.05).

FC log2	Protein	Gene name	UPID	p-value	q-value
2.22	Haptoglobin	HP	P00738	1.52E-03	7.59E-03
1.11	Complement component C9	C9	P02748	2.13E-04	2.42E-03
1.09	Complement component C8 gamma chain	C8G	P07360	4.21E-05	9.77E-04
0.95	Histidine-rich glycoprotein	HRG	P04196	3.77E-03	1.73E-02
0.85	Apolipoprotein B-100	APOB	P04114	1.62E-04	2.11E-03
0.82	Alpha-1-acid glycoprotein 1	ORM1	P02763	6.02E-04	5.11E-03
0.80	Inter-alpha-trypsin inhibitor heavy chain H3	ITIH3	Q06033	6.81E-07	5.79E-05
0.75	Adipocyte plasma membrane-associated protein	APMAP	Q9HDC9	1.08E-02	4.08E-02
0.67	Hyaluronan-binding protein 2	HABP2	Q14520	1.35E-02	4.58E-02
0.65	Alpha-1-antichymotrypsin	SERPINA3	P01011	1.44E-03	7.42E-03
0.59	Complement factor B	CFB	P00751	4.08E-09	6.94E-07
0.55	Complement component C8 alpha chain	C8A	P07357	9.43E-06	5.34E-04
0.46	Complement component C8 beta chain	C8B	P07358	5.44E-04	4.94E-03
0.46	Complement C1r subcomponent	C1R	P00736	8.93E-05	1.38E-03
0.45	Ceruloplasmin	CP	P00450	2.25E-05	9.57E-04
0.41	Complement factor H-related protein 2	CFHR2	P36980	1.54E-02	4.95E-02
0.41	Fibrinogen alpha chain	FGA	P02671	7.06E-04	5.47E-03
0.40	Complement C4-B	C4B	P0C0L5	7.97E-05	1.36E-03
0.40	Serum amyloid P-component	APCS	P02743	1.13E-02	4.08E-02
0.38	Fibrinogen gamma chain	FGG	P02679	1.80E-04	2.18E-03
0.37	Complement component C6	C6	P13671	1.15E-02	4.09E-02
0.34	Complement C5	C5	P01031	1.32E-03	7.23E-03
0.32	Fibrinogen beta chain	FGB	P02675	9.56E-04	6.25E-03
0.29	Complement C1s subcomponent	C1S	P09871	4.60E-05	9.77E-04
0.28	Coagulation factor XIII B chain	F13B	P05160	1.40E-02	4.66E-02
0.28	Vitamin K-dependent protein S	PROS1	P07225	8.46E-03	3.43E-02
-0.21	Serum albumin	ALB	P02768	3.22E-03	1.57E-02

-0.21	Immunoglobulin lambda variable 1-47	IGLV1-47	P01700	3.16E-04	3.36E-03
-0.23	Inter-alpha-trypsin inhibitor heavy chain H2	ITIH2	P19823	1.20E-03	7.06E-03
-0.23	Ig gamma-1 chain C region	IGHG1	P01857	1.39E-03	7.36E-03
-0.25	Immunoglobulin heavy variable 3-15	IGHV3-15	A0A0B4J1V0	7.61E-03	3.15E-02
-0.26	Fibulin-1	FBLN1	P23142	7.08E-04	5.47E-03
-0.26	Immunoglobulin kappa variable 1-5	IGKV1-5	P01602	9.50E-03	3.75E-02
-0.27	Ig kappa chain C region	IGKC	P01834	9.29E-04	6.25E-03
-0.27	Inter-alpha-trypsin inhibitor heavy chain H1	ITIH1	P19827	8.25E-04	5.85E-03
-0.27	Serotransferrin	TF	P02787	3.51E-05	9.77E-04
-0.28	Immunoglobulin kappa variable 3-20	IGKV3-20	P01619	1.12E-04	1.58E-03
-0.29	Immunoglobulin lambda variable 1-51	IGLV1-51	P01701	1.10E-02	4.08E-02
-0.33	Immunoglobulin kappa variable 1-33	IGKV1-33	P01594	1.15E-03	6.97E-03
-0.37	Alpha-2-HS-glycoprotein	AHSG	P02765	6.77E-05	1.28E-03
-0.37	Afamin	AFM	P43652	1.25E-03	7.07E-03
-0.44	Pigment epithelium-derived factor	SERPINF1	P36955	3.41E-03	1.61E-02
-0.45	Immunoglobulin heavy variable 1-2	IGHV1-2	P23083	5.58E-03	2.37E-02
-0.49	Immunoglobulin heavy variable 3-43D	IGHV3-43D	P0DP04	4.55E-04	4.55E-03
-0.49	Lysozyme C	LYZ	P61626	1.31E-02	4.53E-02
-0.53	Cadherin-5	CDH5	P33151	1.10E-02	4.08E-02
-0.62	Peptidase inhibitor 16	PI16	Q6UXB8	5.52E-04	4.94E-03
-0.64	ATPase family AAA domain-containing protein 2	ATAD2	Q6PL18	1.09E-03	6.84E-03
-0.80	Alpha-fetoprotein	AFP	P02771	3.35E-05	9.77E-04
-0.86	Insulin-like growth factor-binding protein 2	IGFBP2	P18065	5.13E-03	2.23E-02
-0.87	Apolipoprotein A-II	APOA2	P02652	1.44E-02	4.72E-02
-1.15	von Willebrand factor	VWF	P04275	7.95E-04	5.85E-03

Supplementary Table 2. Significantly differentiated proteins in the main cohort DOL3 vs DOL0 (q-value < 0.05).

FC log2	Protein	Gene name	UPID	p-value	q-value
2.81	Haptoglobin	HP	P00738	8.35E-04	4.10E-03
2.63	Immunoglobulin J chain	IGJ	P01591	2.06E-05	2.29E-04
1.82	Complement component C9	C9	P02748	1.30E-05	1.67E-04
1.55	Ig mu chain C region	IGHM	P01871	5.36E-08	8.94E-06
1.55	CD5 antigen-like	CD5L	O43866	7.55E-06	1.45E-04
1.39	Complement component C8 gamma chain	C8G	P07360	2.00E-04	1.39E-03
1.38	Histidine-rich glycoprotein	HRG	P04196	5.82E-05	4.86E-04
1.28	Apolipoprotein(a)	LPA	P08519	1.03E-02	2.87E-02
1.17	Alpha-1-acid glycoprotein 1	ORM1	P02763	8.71E-06	1.46E-04
1.02	Haptoglobin-related protein	HPR	P00739	4.30E-03	1.44E-02
1.02	Alpha-1-acid glycoprotein 2	ORM2	P19652	3.01E-05	3.04E-04
0.89	Alpha-1-antichymotrypsin	SERPINA3	P01011	7.03E-06	1.45E-04
0.84	Hyaluronan-binding protein 2	HABP2	Q14520	9.91E-03	2.80E-02
0.79	Complement component C8 alpha chain	C8A	P07357	8.85E-04	4.15E-03
0.71	Complement factor B	CFB	P00751	1.32E-07	1.11E-05
0.67	Coagulation factor XIII B chain	F13B	P05160	2.88E-06	8.01E-05
0.62	Apolipoprotein B-100	APOB	P04114	1.61E-03	6.28E-03
0.62	Complement component C8 beta chain	C8B	P07358	7.09E-07	3.94E-05
0.56	Fibrinogen gamma chain	FGG	P02679	1.96E-05	2.29E-04
0.55	Fibrinogen beta chain	FGB	P02675	1.07E-05	1.49E-04
0.54	Sialic acid-binding Ig-like lectin 16	SIGLEC16	A6NMB1	1.06E-02	2.90E-02
0.53	C4b-binding protein alpha chain	C4BPA	P04003	1.83E-02	4.44E-02
0.53	Serum amyloid A-4 protein	SAA4	P35542	8.66E-03	2.49E-02
0.53	Fibrinogen alpha chain	FGA	P02671	9.49E-05	7.21E-04
0.50	Lactotransferrin	LTF	P02788	4.31E-03	1.44E-02
0.45	Ceruloplasmin	CP	P00450	7.96E-03	2.33E-02
0.43	Complement C1q subcomponent subunit B	C1QB	P02746	1.80E-04	1.31E-03

0.43	Serum amyloid P-component	APCS	P02743	5.87E-03	1.83E-02
0.38	Complement C1r subcomponent	C1R	P00736	3.06E-04	1.81E-03
0.37	Clusterin	CLU	P10909	1.30E-02	3.29E-02
0.37	Apolipoprotein M	APOM	O95445	1.15E-02	3.01E-02
0.34	Vitamin K-dependent protein S	PROS1	P07225	1.17E-02	3.01E-02
0.31	Complement C1s subcomponent	C1S	P09871	2.23E-04	1.43E-03
0.28	Kininogen-1	KNG1	P01042	8.94E-04	4.15E-03
0.25	Inter-alpha-trypsin inhibitor heavy chain H4	ITIH4	Q14624	2.28E-03	8.11E-03
-0.27	Serum albumin	ALB	P02768	1.78E-02	4.38E-02
-0.33	Ig kappa chain C region	IGKC	P01834	3.14E-04	1.81E-03
-0.34	Gelsolin	GSN	P06396	1.62E-03	6.28E-03
-0.42	Ig gamma-1 chain C region	IGHG1	P01857	3.86E-04	2.08E-03
-0.42	Pigment epithelium-derived factor	SERPINF1	P36955	4.41E-03	1.44E-02
-0.43	Alpha-1-antitrypsin	SERPINA1	P01009	1.56E-03	6.28E-03
-0.44	Attractin	ATRN	O75882	5.17E-04	2.70E-03
-0.45	Serotransferrin	TF	P02787	1.23E-06	4.11E-05
-0.45	Immunoglobulin kappa variable 3-20	IGKV3-20	P01619	3.10E-05	3.04E-04
-0.48	Immunoglobulin kappa variable 3-11	IGKV3-11	P04433	3.67E-04	2.04E-03
-0.48	Complement component C7	C7	P10643	1.03E-06	4.11E-05
-0.48	Beta-2-glycoprotein 1	APOH	P02749	7.80E-06	1.45E-04
-0.48	Ig kappa chain V-III region POM	IGKV3-15	P01624	2.16E-04	1.43E-03
-0.49	Immunoglobulin lambda constant 2	IGLC2	P0DOY2	1.25E-03	5.21E-03
-0.51	Alpha-2-HS-glycoprotein	AHSG	P02765	2.08E-03	7.57E-03
-0.52	Ig gamma-4 chain C region	IGHG4	P01861	1.82E-03	6.92E-03
-0.52	Immunoglobulin lambda variable 1-47	IGLV1-47	P01700	5.91E-03	1.83E-02
-0.53	Cathelicidin antimicrobial peptide	CAMP	P49913	9.31E-04	4.17E-03
-0.54	Properdin	CFP	P27918	5.57E-05	4.86E-04
-0.54	Ig gamma-2 chain C region	IGHG2	P01859	3.31E-03	1.15E-02
-0.55	Afamin	AFM	P43652	5.34E-03	1.71E-02
-0.56	Immunoglobulin heavy variable 3-15	IGHV3-15	A0A0B4J1V0	2.08E-03	7.57E-03

-0.57	Immunoglobulin heavy variable 1-18	IGHV1-18	A0A0C4DH31	8.79E-05	6.99E-04
-0.57	Immunoglobulin lambda-like polypeptide 5	IGLL5	B9A064	6.04E-03	1.83E-02
-0.60	Ig heavy chain V-I region HG3	IGHV1-3	P0DP01	8.19E-04	4.10E-03
-0.60	Immunoglobulin kappa variable 3D-20	IGKV3D-20	A0A0C4DH25	7.87E-03	2.33E-02
-0.62	Dynein heavy chain 3, axonemal	DNAH3	Q8TD57	2.03E-02	4.85E-02
-0.62	Ig gamma-3 chain C region	IGHG3	P01860	4.78E-05	4.44E-04
-0.63	ATPase family AAA domain-containing protein 2	ATAD2	Q6PL18	1.21E-03	5.19E-03
-0.68	Angiotensinogen	AGT	P01019	9.79E-06	1.49E-04
-0.90	Ficolin-3	FCN3	O75636	1.09E-02	2.94E-02
-1.31	Neutrophil defensin 3	DEFA3	P59666	1.15E-02	3.01E-02
-1.31	Immunoglobulin lambda variable 3-10	IGLV3-10	A0A075B6K4	9.48E-04	4.17E-03
-1.36	Alpha-fetoprotein	AFP	P02771	2.44E-04	1.51E-03

Supplementary Table 3. Significantly differentiated proteins in the main cohort DOL7 vs DOL0 (q-value < 0.05).

FC log2	Protein.names	Gene	UPID	p-value	q-value
3.17	Haptoglobin	HP	P00738	6.11E-04	1.25E-02
1.39	Complement component C9	C9	P02748	1.93E-03	2.69E-02
0.64	Vitamin K-dependent protein S	PROS1	P07225	2.19E-03	2.69E-02
0.61	Alpha-1-acid glycoprotein 1	ORM1	P02763	5.74E-04	1.25E-02
0.40	Complement C1s subcomponent	C1S	P09871	2.67E-05	1.64E-03
0.37	Complement factor B	CFB	P00751	1.30E-04	5.32E-03
0.37	Alpha-1-antichymotrypsin	SERPINA3	P01011	3.22E-04	9.89E-03
0.28	Ceruloplasmin	CP	P00450	2.00E-03	2.69E-02
-0.33	Ig kappa chain V-I region HK102	IGKV1-5	P01602	1.57E-03	2.69E-02

Supplementary Table 4. Significantly differentiated proteins in the validation cohort DOL1 vs DOL0 (q-value < 0.05).

FC log2	Protein name	Gene	UPID	p-value	q-value
2.64	Histidine-rich glycoprotein	HRG	P04196	1.12E-04	5.19E-03
1.40	Apolipoprotein C-III	APOC3	P02656	7.19E-04	9.08E-03
1.38	Complement component C8 alpha chain	C8A	P07357	7.72E-03	3.72E-02
1.11	Apolipoprotein B-100	APOB	P04114	9.82E-04	1.06E-02
0.84	Inter-alpha-trypsin inhibitor heavy chain H3	ITIH3	Q06033	1.18E-03	1.13E-02
0.60	Apolipoprotein E	APOE	P02649	2.75E-03	2.04E-02
0.59	Complement C1q subcomponent subunit C	C1QC	P02747	2.82E-03	2.04E-02
0.58	Complement factor B	CFB	P00751	3.41E-03	2.22E-02
0.55	Plasma protease C1 inhibitor	SERPING1	P05155	1.81E-03	1.47E-02
0.49	Apolipoprotein M	APOM	O95445	1.22E-03	1.13E-02
0.46	Apolipoprotein D	APOD	P05090	5.77E-04	8.34E-03
0.43	Plasma kallikrein	KLKB1	P03952	5.21E-03	2.94E-02
0.40	Complement C1s subcomponent	C1S	P09871	7.62E-03	3.72E-02
0.38	Ceruloplasmin	CP	P00450	4.09E-03	2.53E-02
0.28	Alpha-2-antiplasmin	SERPINF2	P08697	4.74E-04	8.34E-03
-0.32	Ig gamma-2 chain C region	IGHG2	P01859	4.63E-03	2.73E-02
-0.32	Extracellular matrix protein 1	ECM1	Q16610	9.09E-03	4.07E-02
-0.33	Angiotensinogen	AGT	P01019	1.20E-04	5.19E-03
-0.35	Complement component C7	C7	P10643	5.75E-03	3.11E-02
-0.37	Ig heavy chain V-I region HG3	IGHV1-3	P0DP01	7.68E-04	9.08E-03
-0.37	Ig gamma-4 chain C region	IGHG4	P01861	5.34E-04	8.34E-03
-0.39	Immunoglobulin kappa variable 3-11	IGKV3-11	P04433	4.21E-04	8.34E-03
-0.47	Immunoglobulin kappa variable 3D-20	IGKV3D-20	A0A0C4DH25	1.17E-02	4.90E-02
-0.52	Fibulin-1	FBLN1	P23142	3.16E-03	2.17E-02
-0.54	Immunoglobulin lambda constant 2	IGLC2	P0DOY2	8.60E-03	3.99E-02
-0.55	Immunoglobulin kappa variable 1-33	IGKV1-33	P01594	8.76E-05	5.19E-03

-0.84	Peptidase inhibitor 16	PI16	Q6UXB8	4.32E-04	8.34E-03
-0.88	von Willebrand factor	VWF	P04275	5.46E-04	8.34E-03
-4.81	Serum amyloid A-1 protein	SAA1	P0DJI8	1.14E-02	4.90E-02

Supplementary Table 5. Significant proteins in the validation cohort DOL3 vs DOL0 (q-value < 0.05).

FC log2	Protein name	Gene	UPID	p-value	q-value
2.23	Haptoglobin	HP	P00738	2.39E-03	7.96E-03
1.50	Apolipoprotein B-100	APOB	P04114	9.58E-07	3.86E-05
1.49	Histidine-rich glycoprotein	HRG	P04196	1.93E-02	3.71E-02
1.40	C4b-binding protein alpha chain	C4BPA	P04003	1.15E-04	1.04E-03
1.11	Apolipoprotein C-III	APOC3	P02656	3.23E-04	1.87E-03
1.07	Alpha-1-acid glycoprotein 2	ORM2	P19652	3.55E-04	1.93E-03
1.05	Ig mu chain C region	IGHM	P01871	2.14E-05	3.70E-04
1.05	Complement component C8 beta chain	C8B	P07358	4.31E-08	5.22E-06
1.02	Complement component C8 alpha chain	C8A	P07357	1.26E-04	1.04E-03
0.98	CD5 antigen-like	CD5L	O43866	1.16E-05	2.81E-04
0.97	Hyaluronan-binding protein 2	HABP2	Q14520	6.14E-05	7.44E-04
0.87	Immunoglobulin J chain	IGJ	P01591	1.67E-04	1.12E-03
0.83	Vitamin K-dependent protein S	PROS1	P07225	1.86E-03	6.42E-03
0.83	Apolipoprotein A-I	APOA1	P02647	2.71E-04	1.73E-03
0.76	Complement factor B	CFB	P00751	9.15E-07	3.86E-05
0.76	Hemopexin	HPX	P02790	2.66E-02	4.87E-02
0.64	Apolipoprotein M	APOM	O95445	5.78E-05	7.44E-04
0.60	Complement C1q subcomponent subunit C	C1QC	P02747	1.36E-03	5.02E-03
0.59	Complement C4-B	C4B	P0C0L5	1.55E-04	1.10E-03
0.56	Collagen alpha-1(I) chain	COL1A1	P02452	8.72E-03	2.11E-02
0.55	Plasma protease C1 inhibitor	SERPING1	P05155	6.72E-03	1.77E-02
0.53	Ceruloplasmin	CP	P00450	5.91E-03	1.59E-02
0.47	Complement C5	C5	P01031	9.24E-03	2.19E-02
0.46	Fibrinogen alpha chain	FGA	P02671	1.65E-02	3.38E-02
0.45	Complement C1r subcomponent	C1R	P00736	8.09E-03	2.00E-02
0.41	Fibronectin	FN1	P02751	3.67E-04	1.93E-03
0.39	Vitronectin	VTN	P04004	1.72E-02	3.47E-02
0.36	Complement C1s subcomponent	C1S	P09871	3.04E-03	9.30E-03

0.29	Apolipoprotein D	APOD	P05090	1.03E-02	2.35E-02
0.27	Complement factor H	CFH	P08603	1.28E-04	1.04E-03
0.23	Prothrombin	F2	P00734	3.07E-03	9.30E-03
-0.26	Gelsolin	GSN	P06396	4.94E-03	1.40E-02
-0.29	Ig gamma-4 chain C region	IGHG4	P01861	1.37E-03	5.02E-03
-0.31	Serotransferrin	TF	P02787	2.94E-03	9.30E-03
-0.31	Immunoglobulin kappa variable 3D-20	IGKV3D-20	A0A0C4DH25	1.65E-02	3.38E-02
-0.32	Serum albumin	ALB	P02768	3.14E-05	4.75E-04
-0.32	Immunoglobulin lambda constant 2	IGLC2	P0DOY2	1.83E-03	6.42E-03
-0.33	Immunoglobulin kappa variable 3-11	IGKV3D-11	P04433	1.08E-03	4.36E-03
-0.36	Immunoglobulin heavy variable 3-21	IGHV3-21	A0A0B4J1V1	2.39E-02	4.52E-02
-0.37	Immunoglobulin heavy variable 3-15	IGHV3-15	A0A0B4J1V0	2.43E-03	7.96E-03
-0.39	Immunoglobulin kappa variable 3-15	IGKV3-15	P01624	1.41E-05	2.85E-04
-0.40	Immunoglobulin kappa variable 2-30	IGKV2-30	P06310	3.25E-04	1.87E-03
-0.40	Immunoglobulin kappa variable 3-20	IGKV3-20	P01619	1.20E-02	2.69E-02
-0.43	Ig gamma-3 chain C region	IGHG3	P01860	4.01E-03	1.18E-02
-0.46	Immunoglobulin lambda variable 3-21	IGLV3-21	P80748	1.31E-04	1.04E-03
-0.47	Complement component C7	C7	P10643	7.90E-03	1.99E-02
-0.47	Angiotensinogen	AGT	P01019	5.64E-04	2.63E-03
-0.48	Afamin	AFM	P43652	2.58E-02	4.80E-02
-0.49	Immunoglobulin kappa variable 2-28	IGKV2-28	A0A075B6P5	5.25E-04	2.54E-03
-0.50	Ig kappa chain C region	IGKC	P01834	1.92E-06	5.80E-05
-0.52	Immunoglobulin lambda-like polypeptide 5	IGLL5	B9A064	8.43E-04	3.52E-03
-0.55	Immunoglobulin heavy variable 1-8	IGHV1-8	P0DP01	1.92E-02	3.71E-02
-0.56	Immunoglobulin kappa variable 1-5	IGKV1-5	P01602	7.42E-05	8.16E-04
-0.59	Platelet basic protein	PPBP	P02775	1.56E-02	3.36E-02
-0.69	Ig gamma-1 chain C region	IGHG1	P01857	3.89E-04	1.96E-03
-0.74	Immunoglobulin heavy variable 1-18	IGHV1-18	A0A0C4DH31	9.68E-03	2.25E-02
-0.96	Transthyretin	TTR	P02766	1.80E-02	3.57E-02
-1.20	Actin, cytoplasmic 1	ACTB	P60709	4.99E-03	1.40E-02

-1.25	Immunoglobulin lambda variable 3-16	IGLV3-16	A0A075B6K0	7.36E-03	1.89E-02
-1.30	Alpha-fetoprotein	AFP	P02771	1.38E-04	1.04E-03
-2.92	Neutrophil defensin 3	DEFA3	P59666	6.71E-04	2.90E-03

Supplementary Table 6. Significant proteins in the validation cohort DOL7 vs DOL0 (q-value < 0.05).

UPID	Protein names	GN	missing values				DOL1 vs DOL0			DOL3 vs DOL0			DOL7 vs DOL0		
			DOL0	DOL1	DOL3	DOL7	p-value	FC	q-value	p-value	FC	q-value	p-value	FC	q-value
P0DJI8	Serum amyloid A-1 protein	SAA1	3	1	0	0	1.68E-07	2.85	3.00E-05	8.57E-01	0.19	9.22E-01	8.30E-01	-0.16	8.96E-01
P00738	Haptoglobin	HP	0	0	0	0	1.48E-04	2.16	3.30E-03	1.52E-03	2.22	7.59E-03	8.35E-04	2.81	4.10E-03
P0CF74	Ig lambda-6 chain C region	IGLC6	10	4	3	4	7.45E-01	1.51	8.45E-01	NA	NA	NA	3.05E-01	0.76	4.07E-01
P0DJI9	Serum amyloid A-2 protein	SAA2	9	0	3	5	2.73E-02	1.34	1.10E-01	8.48E-01	-0.21	9.18E-01	NA	NA	NA
P02763	Alpha-1-acid glycoprotein 1	ORM1	0	0	0	0	1.21E-04	1.01	3.07E-03	6.02E-04	0.82	5.11E-03	8.71E-06	1.17	1.46E-04
P04196	Histidine-rich glycoprotein	HRG	0	0	0	0	1.94E-02	0.85	8.85E-02	3.77E-03	0.95	1.73E-02	5.82E-05	1.38	4.86E-04
P02748	Complement component C9	C9	1	0	0	0	1.18E-04	0.81	3.07E-03	2.13E-04	1.11	2.42E-03	1.30E-05	1.82	1.67E-04
P13671	Complement component C6	C6	0	0	0	0	1.48E-03	0.76	1.54E-02	1.15E-02	0.37	4.09E-02	4.30E-02	0.30	9.26E-02
P02743	Serum amyloid P-component	APCS	0	0	0	0	1.94E-05	0.74	1.73E-03	1.13E-02	0.40	4.08E-02	5.87E-03	0.43	1.83E-02
P02750	Leucine-rich alpha-2-glycoprotein	LRG1	9	1	0	0	8.38E-05	0.73	2.99E-03	3.96E-02	0.67	9.47E-02	NA	NA	NA
P02671	Fibrinogen alpha chain	FGA	0	0	0	0	9.88E-04	0.64	1.35E-02	7.06E-04	0.41	5.47E-03	9.49E-05	0.53	7.21E-04
P02656	Apolipoprotein C-III	APOC3	0	0	0	0	1.65E-03	0.59	1.54E-02	8.09E-02	0.34	1.53E-01	9.47E-02	0.45	1.61E-01
P07360	Complement component C8 gamma chain	C8G	0	0	0	0	6.22E-03	0.58	3.95E-02	4.21E-05	1.09	9.77E-04	2.00E-04	1.39	1.39E-03
P01011	Alpha-1-antichymotrypsin	SERPIN A3	0	0	0	0	3.30E-04	0.58	6.33E-03	1.44E-03	0.65	7.42E-03	7.03E-06	0.89	1.45E-04
P02679	Fibrinogen gamma chain	FGG	0	0	0	0	3.73E-03	0.52	2.89E-02	1.80E-04	0.38	2.18E-03	1.96E-05	0.56	2.29E-04
P0DP04			9	2	4	3	3.31E-01	0.51	5.12E-01	4.55E-04	-0.49	4.55E-03	5.47E-02	-0.17	1.07E-01
A0A075B6 I0		IGLV8-61	4	1	1	1	2.71E-01	0.48	4.64E-01	3.01E-02	-0.33	7.76E-02	1.07E-01	-0.46	1.76E-01
P01591	Immunoglobulin J chain	IGJ	4	1	1	0	2.39E-01	0.42	4.29E-01	2.56E-01	0.30	3.67E-01	2.06E-05	2.63	2.29E-04
P01782	Ig heavy chain V-III region DOB	IGHV3-20	3	5	3	0	5.29E-01	0.42	6.89E-01	7.21E-01	-0.28	8.17E-01	8.44E-02	-0.34	1.48E-01
Q6UXB8	Peptidase inhibitor 16	PI16	0	0	0	0	6.25E-02	0.42	1.80E-01	5.52E-04	-0.62	4.94E-03	4.07E-01	-0.35	5.18E-01
P36980	Complement factor H-related protein 2	CFHR2	0	0	0	0	4.58E-02	0.41	1.48E-01	1.54E-02	0.41	4.95E-02	5.37E-01	-0.08	6.48E-01
P19652	Alpha-1-acid glycoprotein 2	ORM2	1	0	0	0	1.06E-01	0.40	2.55E-01	2.65E-02	0.47	6.94E-02	3.01E-05	1.02	3.04E-04
A0A075B6 K4		IGLV3-10	0	0	0	0	5.16E-01	0.38	6.86E-01	4.54E-01	-0.21	5.80E-01	9.48E-04	-1.31	4.17E-03
P02675	Fibrinogen beta chain	FGB	0	0	0	0	1.91E-02	0.37	8.85E-02	9.56E-04	0.32	6.25E-03	1.07E-05	0.55	1.49E-04
Q03591	Complement factor H-related protein 1	CFHR1	5	1	1	0	8.99E-03	0.37	5.16E-02	8.73E-01	0.08	9.27E-01	5.26E-01	0.04	6.41E-01

P00751	Complement factor B	CFB	0	0	0	0	4.16E-05	0.35	2.47E-03	4.08E-09	0.59	6.94E-07	1.32E-07	0.71	1.11E-05
P04114	Apolipoprotein B-100	APOB	0	0	0	0	7.25E-02	0.35	1.93E-01	1.62E-04	0.85	2.11E-03	1.61E-03	0.62	6.28E-03
P00450	Ceruloplasmin	CP	0	0	0	0	9.27E-04	0.33	1.35E-02	2.25E-05	0.45	9.57E-04	7.96E-03	0.45	2.33E-02
P49755	Transmembrane emp24 domain-containing protein 10	TMED10	13	5	6	8	7.84E-01	0.31	8.62E-01	NA	NA	NA	NA	NA	NA
P15169	Carboxypeptidase N catalytic chain	CPN1	0	0	0	0	1.20E-01	0.30	2.71E-01	5.38E-01	0.01	6.72E-01	6.61E-01	-0.02	7.51E-01
P07358	Complement component C8 beta chain	C8B	0	0	0	0	1.09E-01	0.29	2.58E-01	5.44E-04	0.46	4.94E-03	7.09E-07	0.62	3.94E-05
Q16610	Extracellular matrix protein 1	ECM1	0	0	0	0	2.70E-02	0.28	1.10E-01	3.75E-01	-0.15	5.06E-01	6.79E-01	-0.10	7.66E-01
P18065	Insulin-like growth factor-binding protein 2	IGFBP2	7	4	4	5	5.43E-01	0.27	6.90E-01	5.13E-03	-0.86	2.23E-02	NA	NA	NA
P21453	Sphingosine 1-phosphate receptor 1	S1PR1	13	3	5	2	1.97E-03	0.26	1.75E-02	NA	NA	NA	8.67E-01	0.01	9.19E-01
Q06033	Inter-alpha-trypsin inhibitor heavy chain H3	ITIH3	0	0	0	0	2.30E-01	0.24	4.18E-01	6.81E-07	0.80	5.79E-05	9.35E-02	0.43	1.61E-01
P05160	Coagulation factor XIII B chain	F13B	0	0	0	0	2.08E-01	0.23	3.86E-01	1.40E-02	0.28	4.66E-02	2.88E-06	0.67	8.01E-05
P01344	Insulin-like growth factor II	IGF2	7	1	0	1	1.42E-01	0.22	2.97E-01	6.56E-02	-0.60	1.30E-01	7.75E-01	0.09	8.46E-01
P35542	Serum amyloid A-4 protein	SAA4	0	0	0	0	2.14E-01	0.20	3.93E-01	6.44E-01	0.09	7.65E-01	8.66E-03	0.53	2.49E-02
Q12805	EGF-containing fibulin-like extracellular matrix protein 1	EFEMP1	10	3	3	0	6.77E-02	0.20	1.88E-01	2.84E-01	-0.15	3.99E-01	1.16E-01	-0.36	1.84E-01
P25311	Zinc-alpha-2-glycoprotein	AZGP1	0	0	0	0	1.88E-01	0.20	3.60E-01	1.37E-01	0.18	2.21E-01	8.69E-01	-0.02	9.19E-01
P06681	Complement C2	C2	0	0	0	0	2.93E-01	0.17	4.78E-01	2.01E-02	0.24	5.78E-02	1.93E-01	0.24	2.75E-01
P00736	Complement C1r subcomponent	C1R	0	0	0	0	1.29E-01	0.17	2.83E-01	8.93E-05	0.46	1.38E-03	3.06E-04	0.38	1.81E-03
P02647	Apolipoprotein A-I	APOA1	0	0	0	0	2.91E-01	0.17	4.78E-01	1.39E-01	-0.32	2.24E-01	2.24E-01	0.35	3.12E-01
P06310	Ig kappa chain V-II region RPMI 6410	IGKV2D-30	0	1	0	0	6.94E-01	0.15	8.08E-01	6.09E-02	-0.16	1.28E-01	2.88E-01	-0.17	3.92E-01
P07357	Complement component C8 alpha chain	C8A	0	0	0	0	1.23E-01	0.15	2.73E-01	9.43E-06	0.55	5.34E-04	8.85E-04	0.79	4.15E-03
P36955	Pigment epithelium-derived factor	SERPINF1	0	0	0	0	1.85E-01	0.15	3.60E-01	3.41E-03	-0.44	1.61E-02	4.41E-03	-0.42	1.44E-02
P33151	Cadherin-5	CDH5	2	1	0	0	3.17E-01	0.15	4.99E-01	1.10E-02	-0.53	4.08E-02	5.85E-02	-0.58	1.14E-01
P61769	Beta-2-microglobulin	B2M	0	0	0	0	5.57E-01	0.14	7.00E-01	9.27E-01	0.06	9.61E-01	5.49E-01	0.23	6.50E-01

P0DOY3			0	0	0	0	2.59E-01	0.13	4.61E-01	9.24E-01	0.11	9.61E-01	4.11E-02	-0.62	9.16E-02
P27918	Properdin	CFP	0	0	0	0	5.11E-01	0.13	6.83E-01	8.72E-02	-0.25	1.53E-01	5.57E-05	-0.54	4.86E-04
Q9NZP8	Complement C1r subcomponent-like protein	C1RL	3	1	0	1	6.32E-01	0.10	7.76E-01	4.11E-02	0.29	9.71E-02	4.79E-01	0.17	5.88E-01
P0C0L5	Complement C4-B	C4B	0	0	0	0	9.47E-02	0.09	2.34E-01	7.97E-05	0.40	1.36E-03	2.68E-02	0.30	6.14E-02
P02652	Apolipoprotein A-II	APOA2	0	0	0	0	5.77E-01	0.09	7.18E-01	1.44E-02	-0.87	4.72E-02	6.00E-02	0.83	1.15E-01
P01031	Complement C5	C5	0	0	0	0	3.62E-01	0.09	5.46E-01	1.32E-03	0.34	7.23E-03	7.11E-02	0.25	1.31E-01
O14791	Apolipoprotein L1	APOL1	0	0	0	0	8.60E-01	0.09	8.88E-01	3.39E-02	0.59	8.36E-02	3.61E-01	0.26	4.70E-01
P02747	Complement C1q subcomponent subunit C	C1QC	0	0	0	0	2.64E-01	0.09	4.64E-01	1.64E-02	0.25	5.16E-02	2.57E-02	0.33	5.96E-02
P01718	Ig lambda chain V-IV region Kern		10	0	1	2	8.64E-01	0.08	8.88E-01	NA	NA	NA	1.00E+00	0.79	1.00E+00
P80748	Ig lambda chain V-III region LOI	IGLV3-9	0	1	0	0	8.78E-01	0.07	8.93E-01	3.28E-02	-0.64	8.21E-02	9.51E-01	-0.02	9.69E-01
P48357	Leptin receptor	LEPR	0	0	0	0	2.86E-01	0.07	4.76E-01	3.57E-01	-0.03	4.85E-01	1.74E-02	-0.19	4.33E-02
P0DOY2			0	0	0	0	7.26E-01	0.07	8.37E-01	1.30E-01	-0.17	2.14E-01	1.25E-03	-0.49	5.21E-03
P01024	Complement C3	C3	0	0	0	0	8.79E-02	0.06	2.27E-01	2.37E-01	0.03	3.48E-01	2.96E-01	0.10	3.99E-01
P09871	Complement C1s subcomponent	C1S	0	0	0	0	4.85E-01	0.06	6.69E-01	4.60E-05	0.29	9.77E-04	2.23E-04	0.31	1.43E-03
P10643	Complement component C7	C7	0	0	0	0	4.99E-01	0.05	6.77E-01	4.61E-03	-0.19	2.06E-02	1.03E-06	-0.48	4.11E-05
P01706	Ig lambda chain V-II region BOH		3	3	0	0	4.12E-01	0.04	6.01E-01	4.33E-01	-0.19	5.57E-01	6.44E-01	-0.08	7.42E-01
P01766	Ig heavy chain V-III region BRO		15	3	5	5	8.53E-01	0.04	8.88E-01	NA	NA	NA	NA	NA	NA
P01859	Ig gamma-2 chain C region	IGHG2	0	0	0	0	9.24E-01	0.04	9.35E-01	4.80E-01	-0.08	6.08E-01	3.31E-03	-0.54	1.15E-02
P05156	Complement factor I	CFI	0	0	0	0	8.11E-01	0.04	8.74E-01	9.80E-01	-0.06	9.92E-01	1.56E-01	0.19	2.41E-01
P00739	Haptoglobin-related protein	HPR	5	2	0	1	9.44E-01	0.03	9.49E-01	1.54E-01	0.36	2.44E-01	4.30E-03	1.02	1.44E-02
P26927	Hepatocyte growth factor-like protein	MST1	0	0	0	0	8.70E-01	0.03	8.90E-01	3.23E-01	0.07	4.46E-01	2.49E-01	-0.13	3.41E-01
P04003	C4b-binding protein alpha chain	C4BPA	0	0	0	0	8.60E-01	0.03	8.88E-01	8.19E-02	0.23	1.53E-01	1.83E-02	0.53	4.44E-02
P02766	Transthyretin	TTR	0	0	0	0	5.37E-01	0.02	6.89E-01	1.79E-02	-0.24	5.43E-02	3.16E-01	-0.12	4.19E-01
P02768	Serum albumin	ALB	0	0	0	0	3.75E-01	0.02	5.61E-01	3.22E-03	-0.21	1.57E-02	1.78E-02	-0.27	4.38E-02
P01042	Kininogen-1	KNG1	0	0	0	0	7.35E-01	0.02	8.39E-01	9.25E-02	0.12	1.57E-01	8.94E-04	0.28	4.15E-03
P01009	Alpha-1-antitrypsin	SERPIN A1	0	0	0	0	5.38E-01	0.01	6.89E-01	7.52E-01	-0.01	8.47E-01	1.56E-03	-0.43	6.28E-03
P61626	Lysozyme C	LYZ	0	0	2	3	9.53E-01	0.00	9.53E-01	1.31E-02	-0.49	4.53E-02	4.06E-01	-0.20	5.18E-01
P06396	Gelsolin	GSN	0	0	0	0	7.29E-01	-0.01	8.37E-01	4.23E-01	-0.05	5.49E-01	1.62E-03	-0.34	6.28E-03
P04217	Alpha-1B-glycoprotein	A1BG	0	0	0	0	5.38E-01	-0.01	6.89E-01	4.15E-01	-0.06	5.47E-01	9.22E-02	0.13	1.60E-01
P00734	Prothrombin	F2	0	0	0	0	8.07E-01	-0.01	8.74E-01	2.15E-01	0.11	3.23E-01	6.06E-01	0.04	7.08E-01
P01008	Antithrombin-III	SERPIN C1	0	0	0	0	6.87E-01	-0.02	8.07E-01	6.91E-01	0.01	7.94E-01	1.14E-01	0.10	1.83E-01

Q14624	Inter-alpha-trypsin inhibitor heavy chain H4	ITIH4	0	0	0	0	6.90E-01	-0.02	8.07E-01	2.01E-02	0.21	5.78E-02	2.28E-03	0.25	8.11E-03
P02746	Complement C1q subcomponent subunit B	C1QB	0	0	0	0	7.81E-01	-0.02	8.62E-01	3.62E-02	0.19	8.80E-02	1.80E-04	0.43	1.31E-03
P02774	Vitamin D-binding protein	GC	0	0	0	0	8.15E-01	-0.03	8.74E-01	2.40E-01	-0.10	3.49E-01	6.56E-01	0.05	7.51E-01
P02749	Beta-2-glycoprotein 1	APOH	0	0	0	0	4.26E-01	-0.03	6.12E-01	8.28E-02	-0.21	1.53E-01	7.80E-06	-0.48	1.45E-04
A0A075B6I9	Ig lambda chain V region 4A	IGLV7-46	0	0	0	0	5.87E-01	-0.04	7.25E-01	5.76E-01	-0.22	7.04E-01	9.83E-01	-0.02	9.89E-01
P02787	Serotransferrin	TF	0	0	0	0	4.37E-01	-0.04	6.18E-01	3.51E-05	-0.27	9.77E-04	1.23E-06	-0.45	4.11E-05
P01019	Angiotensinogen	AGT	0	0	0	0	1.60E-01	-0.04	3.20E-01	5.43E-01	-0.09	6.74E-01	9.79E-06	-0.68	1.49E-04
O43196	MutS protein homolog 5	MSH5	0	1	0	1	5.58E-01	-0.04	7.00E-01	9.01E-02	-0.33	1.55E-01	4.16E-01	-0.25	5.26E-01
P01860	Ig gamma-3 chain C region	IGHG3	0	0	0	0	8.30E-01	-0.05	8.79E-01	4.30E-02	-0.19	9.74E-02	4.78E-05	-0.62	4.44E-04
P05090	Apolipoprotein D	APOD	0	0	0	0	5.01E-02	-0.05	1.58E-01	4.44E-02	0.11	9.81E-02	1.29E-01	0.18	2.01E-01
P00747	Plasminogen	PLG	0	0	0	0	2.70E-01	-0.05	4.64E-01	5.87E-01	-0.07	7.13E-01	6.78E-02	-0.16	1.29E-01
P02765	Alpha-2-HS-glycoprotein	AHSG	0	0	0	0	3.56E-01	-0.05	5.42E-01	6.77E-05	-0.37	1.28E-03	2.08E-03	-0.51	7.57E-03
P01861	Ig gamma-4 chain C region	IGHG4	0	0	0	0	3.44E-01	-0.06	5.27E-01	3.85E-01	-0.12	5.16E-01	1.82E-03	-0.52	6.92E-03
Q96IY4	Carboxypeptidase B2	CPB2	2	0	0	0	6.40E-01	-0.06	7.80E-01	6.77E-01	-0.01	7.83E-01	7.56E-01	0.06	8.41E-01
P08697	Alpha-2-antiplasmin	SERPINF2	0	0	0	0	1.71E-01	-0.06	3.37E-01	4.38E-02	0.11	9.80E-02	4.32E-02	-0.20	9.26E-02
P08603	Complement factor H	CFH	0	0	0	0	1.51E-01	-0.07	3.06E-01	8.73E-01	0.00	9.27E-01	4.93E-02	0.15	1.00E-01
P07195	L-lactate dehydrogenase B chain	LDHB	3	1	1	1	7.65E-01	-0.07	8.51E-01	2.16E-02	-0.70	6.01E-02	8.93E-01	0.03	9.38E-01
Q14520	Hyaluronan-binding protein 2	HABP2	0	0	0	0	7.51E-01	-0.07	8.46E-01	1.35E-02	0.67	4.58E-02	9.91E-03	0.84	2.80E-02
A0A0B4J1V0		IGHV3-15	0	0	0	0	3.27E-01	-0.08	5.10E-01	7.61E-03	-0.25	3.15E-02	2.08E-03	-0.56	7.57E-03
P02771	Alpha-fetoprotein	AFP	0	0	0	0	8.26E-01	-0.08	8.79E-01	3.35E-05	-0.80	9.77E-04	2.44E-04	-1.36	1.51E-03
P04004	Vitronectin	VTN	0	0	0	0	6.68E-02	-0.08	1.88E-01	9.03E-01	0.01	9.47E-01	2.35E-02	0.18	5.52E-02
P01023	Alpha-2-macroglobulin	A2M	0	0	0	0	4.43E-03	-0.09	3.28E-02	8.82E-01	-0.02	9.31E-01	9.39E-01	0.01	9.68E-01
P07225	Vitamin K-dependent protein S	PROS1	1	0	0	0	5.30E-01	-0.09	6.89E-01	8.46E-03	0.28	3.43E-02	1.17E-02	0.34	3.01E-02
P01594	Ig kappa chain V-I region AU		0	0	0	0	8.55E-01	-0.09	8.88E-01	1.15E-03	-0.33	6.97E-03	2.10E-01	-0.29	2.94E-01
P03952	Plasma kallikrein	KLKB1	0	0	0	0	9.28E-02	-0.10	2.33E-01	4.20E-01	-0.05	5.49E-01	9.51E-01	0.00	9.69E-01
P01703	Ig lambda chain V-I region NEWM		3	0	0	0	1.41E-01	-0.10	2.97E-01	3.15E-01	-0.27	4.38E-01	1.92E-01	-0.20	2.75E-01

P00748	Coagulation factor XII	F12	0	0	0	1	6.52E-01	-0.11	7.90E-01	1.83E-02	-0.71	5.46E-02	4.76E-02	0.03	9.81E-02
A0A075B6P5	Ig kappa chain V-II region FR	IGKV2D-28	1	0	0	0	8.15E-01	-0.12	8.74E-01	1.10E-01	-0.17	1.83E-01	1.08E-01	-0.27	1.76E-01
P02649	Apolipoprotein E	APOE	0	0	0	0	6.85E-01	-0.12	8.07E-01	7.08E-01	0.13	8.07E-01	7.60E-01	0.10	8.41E-01
A0A075B6H9		IGLV4-69	12	4	2	5	4.15E-01	-0.12	6.01E-01	NA	NA	NA	NA	NA	NA
P49747	Cartilage oligomeric matrix protein	COMP	1	0	0	0	3.99E-01	-0.13	5.87E-01	1.93E-01	-0.30	2.93E-01	3.33E-01	-0.15	4.38E-01
O95445	Apolipoprotein M	APOM	0	0	0	0	5.06E-02	-0.13	1.58E-01	5.19E-01	-0.11	6.54E-01	1.15E-02	0.37	3.01E-02
P02751	Fibronectin	FN1	0	0	0	0	3.90E-01	-0.13	5.78E-01	5.91E-01	-0.09	7.13E-01	8.42E-02	0.38	1.48E-01
O75882	Attractin	ATRN	0	0	0	0	1.51E-01	-0.14	3.06E-01	9.92E-01	-0.02	9.92E-01	5.17E-04	-0.44	2.70E-03
P01700	Ig lambda chain V-I region HA		0	0	0	0	2.81E-01	-0.14	4.76E-01	3.16E-04	-0.21	3.36E-03	5.91E-03	-0.52	1.83E-02
Q6PL18	ATPase family AAA domain-containing protein 2	ATAD2	0	0	0	0	5.02E-01	-0.14	6.77E-01	1.09E-03	-0.64	6.84E-03	1.21E-03	-0.63	5.19E-03
P01619	Ig kappa chain V-III region B6		0	0	0	0	2.05E-01	-0.14	3.85E-01	1.12E-04	-0.28	1.58E-03	3.10E-05	-0.45	3.04E-04
P02775	Platelet basic protein	PPBP	0	0	0	0	4.65E-01	-0.15	6.47E-01	2.81E-01	-0.23	3.98E-01	8.37E-01	0.02	8.96E-01
P63104	14-3-3 protein zeta/delta	YWHAZ	12	1	4	5	7.62E-01	-0.15	8.51E-01	NA	NA	NA	NA	NA	NA
A0A0C4DH72		IGKV1-6	8	3	0	4	3.08E-01	-0.15	4.90E-01	1.77E-02	-0.57	5.43E-02	4.02E-01	-0.15	5.18E-01
P01834	Ig kappa chain C region	IGKC	0	0	0	0	8.61E-03	-0.15	5.11E-02	9.29E-04	-0.27	6.25E-03	3.14E-04	-0.33	1.81E-03
P05155	Plasma protease C1 inhibitor	SERPING1	1	0	0	0	6.82E-01	-0.16	8.07E-01	8.45E-01	0.11	9.18E-01	1.77E-01	-0.43	2.62E-01
P02753	Retinol-binding protein 4	RBP4	0	0	0	0	4.39E-01	-0.16	6.18E-01	9.66E-01	0.00	9.84E-01	5.40E-01	0.11	6.48E-01
A0A0B4J1V1	Ig heavy chain V-III region JON	IGHV3-21	0	0	0	0	9.17E-02	-0.16	2.33E-01	4.07E-01	-0.19	5.40E-01	7.83E-02	-0.37	1.41E-01
P02790	Hemopexin	HPX	0	0	0	0	3.87E-02	-0.17	1.33E-01	9.96E-02	0.30	1.68E-01	4.39E-02	0.90	9.27E-02
P02760	Protein AMBP	AMBP	0	0	0	0	6.04E-03	-0.17	3.95E-02	6.31E-02	-0.14	1.28E-01	8.31E-01	0.00	8.96E-01
A0A0C4DH25		IGKV3D-20	0	0	0	0	2.18E-02	-0.17	9.44E-02	1.31E-01	-0.14	2.14E-01	7.87E-03	-0.60	2.33E-02
P19827	Inter-alpha-trypsin inhibitor heavy chain H1	ITIH1	0	0	0	0	1.64E-03	-0.18	1.54E-02	8.25E-04	-0.27	5.85E-03	1.77E-01	-0.10	2.62E-01
P04433	Ig kappa chain V-III region VG	IGKV3D-11	0	0	0	0	3.09E-03	-0.18	2.50E-02	5.04E-02	-0.19	1.08E-01	3.67E-04	-0.48	2.04E-03
P10909	Clusterin	CLU	0	0	0	0	2.03E-01	-0.20	3.85E-01	6.72E-01	0.08	7.83E-01	1.30E-02	0.37	3.29E-02
P01602	Ig kappa chain V-I region HK102	IGKV1-5	0	0	0	0	1.19E-01	-0.20	2.71E-01	9.50E-03	-0.26	3.75E-02	1.61E-01	-0.33	2.44E-01
Q8TD57	Dynein heavy chain 3, axonemal	DNAH3	5	2	2	2	3.67E-02	-0.20	1.28E-01	2.51E-02	-0.15	6.89E-02	2.03E-02	-0.62	4.85E-02
P23142	Fibulin-1	FBLN1	0	0	0	0	4.84E-03	-0.20	3.44E-02	7.08E-04	-0.26	5.47E-03	7.38E-02	-0.25	1.34E-01
O43866	CD5 antigen-like	CD5L	2	1	0	0	2.68E-01	-0.21	4.64E-01	6.61E-01	-0.13	7.80E-01	7.54E-06	1.55	1.45E-04

P01704	Ig lambda chain V-II region TOG		3	1	0	1	6.81E-01	-0.22	8.07E-01	6.00E-01	-0.05	7.18E-01	7.69E-01	-0.06	8.45E-01
P0DP01	Ig heavy chain V-I region HG3	IGHV1-3	0	0	0	0	3.27E-02	-0.24	1.24E-01	4.19E-02	-0.28	9.74E-02	8.19E-04	-0.60	4.10E-03
P01624	Ig kappa chain V-III region POM		0	0	0	0	3.56E-04	-0.26	6.33E-03	8.31E-02	-0.17	1.53E-01	2.16E-04	-0.48	1.43E-03
P01857	Ig gamma-1 chain C region	IGHG1	0	0	0	0	2.68E-03	-0.26	2.27E-02	1.39E-03	-0.23	7.36E-03	3.86E-04	-0.42	2.08E-03
P00746	Complement factor D	CFD	2	0	0	1	1.34E-01	-0.27	2.87E-01	2.65E-02	-0.28	6.94E-02	5.00E-02	-0.33	1.01E-01
P01871	Ig mu chain C region	IGHM	0	0	0	0	7.44E-03	-0.27	4.57E-02	6.60E-02	0.17	1.30E-01	5.36E-08	1.55	8.94E-06
P49913	Cathelicidin antimicrobial peptide	CAMP	0	0	0	0	4.41E-01	-0.27	6.18E-01	1.93E-01	-0.30	2.93E-01	9.31E-04	-0.53	4.17E-03
P01701	Ig lambda chain V-I region NEW		0	0	0	0	6.05E-02	-0.28	1.76E-01	1.10E-02	-0.29	4.08E-02	6.97E-02	-0.26	1.29E-01
P19823	Inter-alpha-trypsin inhibitor heavy chain H2	ITIH2	0	0	0	0	6.27E-04	-0.30	1.01E-02	1.20E-03	-0.23	7.06E-03	2.35E-01	-0.11	3.24E-01
P01709	Ig lambda chain V-II region MGC		0	0	0	0	1.42E-03	-0.30	1.54E-02	8.70E-02	-0.28	1.53E-01	5.91E-01	-0.12	6.95E-01
P43652	Afamin	AFM	0	0	0	0	6.06E-03	-0.30	3.95E-02	1.25E-03	-0.37	7.07E-03	5.34E-03	-0.55	1.71E-02
A0A0C4D H31		IGHV1-18	0	0	0	0	1.76E-02	-0.30	8.49E-02	3.15E-02	-0.20	7.99E-02	8.79E-05	-0.57	6.99E-04
P07437	Tubulin beta chain	TUBB	9	4	6	5	3.08E-01	-0.31	4.90E-01	NA	NA	NA	NA	NA	NA
B9A064	Immunoglobulin lambda-like polypeptide 5	IGLL5	0	0	0	0	1.60E-03	-0.31	1.54E-02	2.61E-02	-0.15	6.94E-02	6.04E-03	-0.57	1.83E-02
P04275	von Willebrand factor	VWF	0	0	0	0	3.01E-01	-0.32	4.87E-01	7.95E-04	-1.15	5.85E-03	6.97E-02	-0.56	1.29E-01
O75636	Ficolin-3	FCN3	2	0	0	1	5.00E-01	-0.34	6.77E-01	8.60E-02	-0.82	1.53E-01	1.09E-02	-0.90	2.94E-02
Q96PD5	N-acetylmuramoyl-L-alanine amidase	PGLYRP 2	0	0	0	0	7.08E-05	-0.34	2.99E-03	5.72E-02	-0.29	1.22E-01	4.55E-01	-0.08	5.67E-01
A0M8Q6	Ig lambda-7 chain C region	IGLC7	8	3	3	5	2.81E-02	-0.37	1.11E-01	6.34E-02	-0.18	1.28E-01	NA	NA	NA
Q04756	Hepatocyte growth factor activator	HGFAC	3	0	1	0	4.32E-02	-0.37	1.42E-01	7.92E-01	-0.08	8.86E-01	5.48E-01	0.09	6.50E-01
P08519	Apolipoprotein(a)	LPA	0	1	0	1	1.87E-01	-0.38	3.60E-01	1.89E-01	0.55	2.92E-01	1.03E-02	1.28	2.87E-02
P23083	Ig heavy chain V-I region V35		0	0	0	0	3.47E-02	-0.39	1.28E-01	5.58E-03	-0.45	2.37E-02	1.93E-01	-0.27	2.75E-01
A0A0B4J1 X5		IGHV3-74	1	0	2	1	1.76E-02	-0.41	8.49E-02	8.02E-02	-0.21	1.53E-01	1.05E-01	-0.38	1.75E-01
P01034	Cystatin-C	CST3	4	1	3	1	1.23E-02	-0.41	6.61E-02	8.12E-02	-0.27	1.53E-01	5.15E-02	-0.36	1.02E-01
P02452	Collagen alpha-1(I) chain	COL1A1	1	3	0	0	1.19E-02	-0.42	6.61E-02	2.19E-01	-0.10	3.26E-01	4.19E-02	0.34	9.21E-02
P60709	Actin, cytoplasmic 1	ACTB	0	0	0	0	2.05E-02	-0.42	9.13E-02	9.36E-01	-0.04	9.64E-01	1.94E-01	-0.23	2.75E-01
A0A0B4J1 U7		IGHV6-1	1	1	0	0	3.61E-02	-0.42	1.28E-01	2.57E-01	-0.31	3.67E-01	1.27E-01	-0.49	2.00E-01
Q15485	Ficolin-2	FCN2	12	2	2	4	1.10E-01	-0.49	2.58E-01	8.34E-01	-0.02	9.18E-01	1.03E-01	-0.35	1.74E-01

Q08380	Galectin-3-binding protein	LGALS3 BP	0	3	6	0	6.90E-02	-0.49	1.89E-01	NA	NA	NA	4.26E-01	0.24	5.35E-01
P04406	Glyceraldehyde-3-phosphate dehydrogenase	GAPDH	16	5	4	7	5.49E-02	-0.52	1.69E-01	NA	NA	NA	NA	NA	NA
P27169	Serum paraoxonase/arylesterase 1	PON1	1	1	0	0	1.46E-01	-0.53	3.01E-01	9.63E-01	-0.03	9.84E-01	1.87E-01	-0.29	2.74E-01
A0A075B6 J9		IGLV2-18	7	1	5	6	2.85E-01	-0.54	4.76E-01	NA	NA	NA	NA	NA	NA
P02788	Lactotransferrin	LTF	0	0	0	0	5.68E-02	-0.55	1.71E-01	1.75E-01	-0.35	2.76E-01	4.31E-03	0.50	1.44E-02
P05106	Integrin beta-3	ITGB3	11	3	6	4	1.18E-01	-0.55	2.71E-01	NA	NA	NA	4.45E-02	-0.28	9.29E-02
P21333	Filamin-A	FLNA	4	0	1	1	1.34E-01	-0.66	2.87E-01	8.40E-01	0.09	9.18E-01	7.20E-01	-0.16	8.07E-01
P62937	Peptidyl-prolyl cis-trans isomerase A	PPIA	13	1	7	8	3.68E-02	-0.79	1.28E-01	NA	NA	NA	NA	NA	NA
P69892	Hemoglobin subunit gamma-2	HBG2	0	0	0	0	6.03E-02	-0.81	1.76E-01	9.90E-01	-0.07	9.92E-01	4.76E-01	-0.26	5.88E-01
P68871	Hemoglobin subunit beta	HBB	0	0	0	0	9.67E-02	-0.90	2.36E-01	5.71E-01	0.23	7.03E-01	9.79E-01	-0.05	9.89E-01
P69905	Hemoglobin subunit alpha	HBA1	0	0	0	0	7.26E-02	-0.91	1.93E-01	8.35E-01	0.08	9.18E-01	9.05E-01	-0.02	9.45E-01
Q99879	Histone H2B type 1-M	HIST1H2 BM	11	3	7	10	4.32E-02	-0.93	1.42E-01	NA	NA	NA	NA	NA	NA
P10720	Platelet factor 4 variant	PF4V1	2	1	1	1	2.66E-02	-0.95	1.10E-01	2.29E-01	0.33	3.39E-01	2.80E-02	-0.66	6.32E-02
P35443	Thrombospondin-4	THBS4	4	3	3	2	1.61E-03	-0.99	1.54E-02	8.90E-02	-0.41	1.54E-01	1.13E-01	-0.41	1.83E-01
P69891	Hemoglobin subunit gamma-1	HBG1	1	0	1	0	3.05E-02	-1.21	1.18E-01	4.28E-02	0.39	9.74E-02	1.58E-01	-0.65	2.42E-01
P59666	Neutrophil defensin 3	DEFA3	0	0	0	0	8.65E-02	-1.25	2.26E-01	8.49E-02	-0.79	1.53E-01	1.15E-02	-1.31	3.01E-02
Q99878	Histone H2A type 1-J	HIST1H2 AJ	6	1	5	6	1.52E-02	-1.47	7.74E-02	NA	NA	NA	NA	NA	NA
P30043	Flavin reductase (NADPH)	BLVRB	3	0	2	1	1.32E-02	-2.24	6.90E-02	6.28E-02	0.87	1.28E-01	9.30E-01	-0.11	9.65E-01
P16112	Aggrecan core protein	ACAN	13	4	6	6	NA	NA	NA	NA	NA	NA	NA	NA	NA
Q9HDC9	Adipocyte plasma membrane-associated protein	APMAP	15	4	3	5	NA	NA	NA	1.08E-02	0.75	4.08E-02	NA	NA	NA
P02745	Complement C1q subcomponent subunit A	C1QA	19	9	4	5	NA	NA	NA	NA	NA	NA	NA	NA	NA
P02458	Collagen alpha-1(II) chain	COL2A1	25	4	7	7	NA	NA	NA	NA	NA	NA	NA	NA	NA
P22792	Carboxypeptidase N subunit 2	CPN2	18	5	4	4	NA	NA	NA	3.41E-01	-0.14	4.68E-01	NA	NA	NA
P12259	Coagulation factor V	F5	15	9	3	4	NA	NA	NA	2.07E-02	0.73	5.88E-02	NA	NA	NA
P00740	Coagulation factor IX	F9	11	6	5	4	NA	NA	NA	NA	NA	NA	6.17E-01	0.05	7.16E-01

B1AL88	Transmembrane protein FAM155A	FAM155A	20	4	5	7	NA	NA	NA	NA	NA	NA	NA	NA	NA
P01876	Ig alpha-1 chain C region	IGHA1	12	8	8	10	NA	NA	NA	NA	NA	NA	NA	NA	NA
A0A0A0MS15		IGHV3-49	19	9	9	4	NA	NA	NA	NA	NA	NA	NA	NA	NA
P0DP09		IGKV1D-13	13	8	6	3	NA	NA	NA	NA	NA	NA	1.73E-01	-0.47	2.60E-01
P15814	Immunoglobulin lambda-like polypeptide 1	IGLL1	13	5	5	6	NA	NA	NA	NA	NA	NA	NA	NA	NA
Q7LBC6	Lysine-specific demethylase 3B	KDM3B	22	4	10	8	NA	NA	NA	NA	NA	NA	NA	NA	NA
P48740	Mannan-binding lectin serine protease 1	MASP1	14	6	6	4	NA	NA	NA	NA	NA	NA	NA	NA	NA
P14618	Pyruvate kinase PKM	PKM	12	8	4	7	NA	NA	NA	6.77E-01	-0.08	7.83E-01	NA	NA	NA
P49908	Selenoprotein P	SEPP1	16	8	1	5	NA	NA	NA	4.81E-02	-0.09	1.05E-01	NA	NA	NA
A6NMB1	Sialic acid-binding Ig-like lectin 16	SIGLEC16	9	1	2	4	NA	NA	NA	8.53E-02	0.26	1.53E-01	1.06E-02	0.54	2.90E-02
Q96RL7	Vacuolar protein sorting-associated protein 13A	VPS13A	14	6	4	4	NA	NA	NA	1.80E-01	-0.29	2.80E-01	NA	NA	NA
P01705	Ig lambda chain V-II region NEI		15	5	4	7	NA	NA	NA	NA	NA	NA	NA	NA	NA

Supplementary Table 7. All proteins in the main cohort sorted by DOL1 fold change. FC: log2(DOLX vs DOL0 fold change difference)

UPID	Protein nnames	GN	missing values				DOL1 vs DOL0			DOL3 vs DOL0			DOL7 vs DOL0		
			DOL0	DOL1	DOL3	DOL7	p-value	FC	q-value	p-value	FC	q-value	p-value	FC	q-value
P00738	Haptoglobin	HP	0	0	0	0	6.11E-04	3.17	1.25E-02	2.06E-02	2.56	7.22E-02	2.39E-03	2.23	7.96E-03
P0DJI9	Serum amyloid A-2 protein	SAA2	4	0	5	6	1.66E-02	2.22	7.74E-02	NA	NA	NA	NA	NA	NA
P0DJI8	Serum amyloid A-1 protein	SAA1	2	0	1	5	5.06E-02	1.87	1.50E-01	1.14E-02	-4.81	4.90E-02	NA	NA	NA
P02679	Fibrinogen gamma chain	FGG	0	0	0	0	7.58E-02	1.86	1.90E-01	8.61E-02	0.28	1.68E-01	9.42E-02	0.33	1.42E-01
P02675	Fibrinogen beta chain	FGB	0	0	0	0	6.31E-02	1.79	1.70E-01	8.81E-02	0.38	1.68E-01	1.79E-01	0.22	2.38E-01
P02671	Fibrinogen alpha chain	FGA	0	0	0	0	4.35E-02	1.73	1.41E-01	4.85E-01	0.28	5.68E-01	1.65E-02	0.46	3.38E-02
P02748	Complement component C9	C9	3	0	0	0	1.93E-03	1.39	2.69E-02	2.84E-01	0.81	3.97E-01	1.17E-01	1.23	1.73E-01
P04003	C4b-binding protein alpha chain	C4BPA	0	0	0	0	4.38E-02	0.98	1.41E-01	9.57E-02	1.02	1.75E-01	1.15E-04	1.40	1.04E-03
O75636	Ficolin-3	FCN3	0	0	0	0	5.53E-03	0.85	5.23E-02	3.51E-01	-0.42	4.61E-01	1.42E-01	-0.48	1.99E-01
P02743	Serum amyloid P-component	APCS	0	0	1	1	6.34E-02	0.70	1.70E-01	2.24E-02	0.54	7.25E-02	7.40E-01	-0.19	8.06E-01
P07225	Vitamin K-dependent protein S	PROS1	0	0	1	0	2.19E-03	0.64	2.69E-02	3.58E-02	0.35	9.31E-02	1.86E-03	0.83	6.42E-03
P02763	Alpha-1-acid glycoprotein 1	ORM1	0	0	0	0	5.74E-04	0.61	1.25E-02	1.86E-01	0.56	2.98E-01	2.87E-02	0.68	5.18E-02
P07357	Complement component C8 alpha chain	C8A	0	0	0	0	1.70E-02	0.60	7.74E-02	7.72E-03	1.38	3.72E-02	1.26E-04	1.02	1.04E-03
P04114	Apolipoprotein B-100	APOB	0	0	0	0	1.64E-01	0.53	3.10E-01	9.82E-04	1.11	1.06E-02	9.58E-07	1.50	3.86E-05
P02656	Apolipoprotein C-III	APOC3	0	0	0	0	9.04E-02	0.46	2.15E-01	7.19E-04	1.40	9.08E-03	3.23E-04	1.11	1.87E-03
P09871	Complement C1s subcomponent	C1S	0	0	0	0	2.67E-05	0.40	1.64E-03	7.62E-03	0.40	3.72E-02	3.04E-03	0.36	9.30E-03
P04196	Histidine-rich glycoprotein	HRG	0	0	0	0	2.91E-01	0.38	4.41E-01	1.12E-04	2.64	5.19E-03	1.93E-02	1.49	3.71E-02
P00751	Complement factor B	CFB	0	0	0	0	1.30E-04	0.37	5.32E-03	3.41E-03	0.58	2.22E-02	9.15E-07	0.76	3.86E-05
P01011	Alpha-1-antichymotrypsin	SERPIN A3	5	0	1	1	3.22E-04	0.37	9.89E-03	NA	NA	NA	NA	NA	NA
P19652	Alpha-1-acid glycoprotein 2	ORM2	0	0	0	0	5.42E-02	0.37	1.51E-01	3.73E-01	0.45	4.80E-01	3.55E-04	1.07	1.93E-03
P35542	Serum amyloid A-4 protein	SAA4	0	0	0	0	3.75E-01	0.34	5.30E-01	5.67E-01	0.49	6.34E-01	4.57E-01	0.62	5.55E-01
P05090	Apolipoprotein D	APOD	0	0	0	0	1.07E-01	0.29	2.40E-01	5.77E-04	0.46	8.34E-03	1.03E-02	0.29	2.35E-02
P01042	Kininogen-1	KNG1	0	0	0	0	2.48E-01	0.29	3.91E-01	3.01E-01	0.16	4.08E-01	7.39E-02	0.33	1.19E-01

P07358	Complement component C8 beta chain	C8B	0	0	0	0	2.41E-01	0.29	3.90E-01	1.68E-02	0.83	6.24E-02	4.31E-08	1.05	5.22E-06
P00748	Coagulation factor XII	F12	0	0	0	0	2.36E-01	0.29	3.90E-01	7.65E-01	0.02	8.22E-01	5.21E-01	-0.32	6.17E-01
P00450	Ceruloplasmin	CP	0	0	0	0	2.00E-03	0.28	2.69E-02	4.09E-03	0.38	2.53E-02	5.91E-03	0.53	1.59E-02
Q03591	Complement factor H-related protein 1	CFHR1	1	1	1	2	2.02E-01	0.26	3.65E-01	6.89E-02	0.67	1.47E-01	NA	NA	NA
P13671	Complement component C6	C6	0	0	0	0	4.62E-02	0.24	1.42E-01	8.47E-01	0.13	8.86E-01	5.37E-02	0.23	9.02E-02
P02747	Complement C1q subcomponent subunit C	C1QC	0	0	0	0	7.53E-03	0.21	5.23E-02	2.82E-03	0.59	2.04E-02	1.36E-03	0.60	5.02E-03
P26927	Hepatocyte growth factor-like protein	MST1	2	0	0	3	4.50E-01	0.21	5.95E-01	2.14E-01	-0.32	3.19E-01	NA	NA	NA
O14791	Apolipoprotein L1	APOL1	5	0	0	0	1.15E-01	0.19	2.53E-01	1.43E-01	0.62	2.47E-01	NA	NA	NA
P01031	Complement C5	C5	0	0	0	0	1.67E-01	0.17	3.11E-01	2.40E-02	0.41	7.25E-02	9.24E-03	0.47	2.19E-02
P0DOY3			0	0	0	1	9.76E-01	0.17	9.79E-01	7.90E-02	-0.22	1.58E-01	1.51E-01	0.81	2.10E-01
P00736	Complement C1r subcomponent	C1R	0	0	0	0	5.29E-01	0.17	6.79E-01	2.44E-01	0.29	3.48E-01	8.09E-03	0.45	2.00E-02
P03952	Plasma kallikrein	KLKB1	2	0	0	0	4.26E-01	0.16	5.76E-01	5.21E-03	0.43	2.94E-02	6.80E-01	0.32	7.62E-01
P02746	Complement C1q subcomponent subunit B	C1QB	0	0	0	0	2.43E-01	0.16	3.90E-01	3.83E-02	0.16	9.77E-02	2.20E-01	0.44	2.86E-01
P01766	Ig heavy chain V-III region BRO		2	1	0	2	9.79E-01	0.15	9.79E-01	1.52E-01	-0.28	2.60E-01	NA	NA	NA
P0C0L5	Complement C4-B	C4B	0	0	0	0	2.15E-02	0.14	9.13E-02	2.92E-02	0.41	7.90E-02	1.55E-04	0.59	1.10E-03
P01019	Angiotensinogen	AGT	0	0	0	0	3.93E-01	0.12	5.44E-01	1.20E-04	-0.33	5.19E-03	5.64E-04	-0.47	2.63E-03
P22792	Carboxypeptidase N subunit 2	CPN2	0	0	0	1	5.02E-01	0.12	6.57E-01	3.31E-02	0.45	8.78E-02	1.35E-01	-0.10	1.92E-01
P02766	Transthyretin	TTR	0	0	0	0	7.29E-01	0.12	8.08E-01	2.16E-01	-0.96	3.19E-01	1.80E-02	-0.96	3.57E-02
Q14624	Inter-alpha-trypsin inhibitor heavy chain H4	ITIH4	0	0	0	0	8.29E-02	0.12	2.04E-01	2.83E-02	-0.16	7.84E-02	1.30E-02	0.16	2.85E-02
P05156	Complement factor I	CFI	0	0	0	0	8.05E-01	0.12	8.61E-01	1.85E-01	0.22	2.98E-01	4.59E-01	0.05	5.55E-01
P01700	Ig lambda chain V-I region HA		0	0	0	0	7.97E-01	0.11	8.61E-01	3.01E-01	-0.16	4.08E-01	2.03E-01	-0.05	2.67E-01
Q06033	Inter-alpha-trypsin inhibitor heavy chain H3	ITIH3	0	0	0	0	2.30E-01	0.10	3.90E-01	1.18E-03	0.84	1.13E-02	6.04E-01	0.11	6.83E-01
P10909	Clusterin	CLU	0	0	0	0	7.99E-01	0.08	8.61E-01	6.49E-02	0.54	1.44E-01	7.53E-01	0.40	8.14E-01
P00734	Prothrombin	F2	0	0	0	0	5.79E-01	0.07	7.19E-01	3.66E-01	-0.03	4.76E-01	3.07E-03	0.23	9.30E-03
P15169	Carboxypeptidase N catalytic chain	CPN1	0	1	0	0	6.38E-01	0.07	7.47E-01	6.59E-02	0.09	1.44E-01	9.41E-01	0.11	9.55E-01
P02647	Apolipoprotein A-I	APOA1	0	0	0	0	7.05E-01	0.06	7.96E-01	5.38E-01	0.00	6.08E-01	2.71E-04	0.83	1.73E-03
Q16610	Extracellular matrix protein 1	ECM1	3	0	1	0	9.59E-01	0.04	9.74E-01	9.09E-03	-0.32	4.07E-02	1.62E-02	0.14	3.38E-02
P10643	Complement component C7	C7	0	0	0	0	3.19E-01	0.04	4.73E-01	5.75E-03	-0.35	3.11E-02	7.90E-03	-0.47	1.99E-02

P01024	Complement C3	C3	0	0	0	0	3.92E-01	0.03	5.44E-01	1.65E-01	0.11	2.76E-01	6.85E-02	0.16	1.12E-01
P27918	Properdin	CFP	1	0	0	1	2.67E-01	0.02	4.10E-01	1.72E-01	-0.35	2.83E-01	4.84E-01	-0.49	5.80E-01
P02790	Hemopexin	HPX	0	0	0	0	8.73E-01	0.01	9.10E-01	2.07E-01	0.37	3.12E-01	2.66E-02	0.76	4.87E-02
P00747	Plasminogen	PLG	0	0	0	0	6.33E-01	0.00	7.47E-01	3.77E-01	-0.05	4.80E-01	8.25E-01	0.03	8.84E-01
P23142	Fibulin-1	FBLN1	0	0	0	0	8.38E-01	-0.02	8.89E-01	3.16E-03	-0.52	2.17E-02	5.81E-01	-0.16	6.63E-01
P02753	Retinol-binding protein 4	RBP4	0	0	0	0	8.82E-01	-0.02	9.11E-01	9.16E-02	0.52	1.72E-01	2.75E-01	0.26	3.51E-01
P08697	Alpha-2-antiplasmin	SERPINF2	0	0	0	0	6.78E-01	-0.03	7.73E-01	4.74E-04	0.28	8.34E-03	9.61E-02	0.00	1.44E-01
P02649	Apolipoprotein E	APOE	0	0	0	0	8.64E-01	-0.04	9.09E-01	2.75E-03	0.60	2.04E-02	9.03E-01	0.16	9.34E-01
P04004	Vitronectin	VTN	0	0	0	0	7.19E-01	-0.04	8.04E-01	8.56E-01	-0.06	8.86E-01	1.72E-02	0.39	3.47E-02
P02749	Beta-2-glycoprotein 1	APOH	0	0	0	0	5.61E-01	-0.05	7.12E-01	1.79E-03	-0.06	1.47E-02	8.10E-02	-0.11	1.27E-01
P36955	Pigment epithelium-derived factor	SERPINF1	1	0	1	0	9.57E-01	-0.06	9.74E-01	6.17E-01	-0.14	6.74E-01	3.55E-01	-0.10	4.43E-01
P02652	Apolipoprotein A-II	APOA2	0	0	0	0	2.56E-02	-0.07	1.02E-01	2.13E-02	0.21	7.25E-02	8.86E-02	0.38	1.37E-01
P01859	Ig gamma-2 chain C region	IGHG2	0	0	0	0	6.65E-01	-0.07	7.66E-01	4.63E-03	-0.32	2.73E-02	8.44E-01	-0.29	8.96E-01
P04433	Ig kappa chain V-III region VG	IGKV3D-11	0	0	0	0	6.15E-01	-0.07	7.41E-01	4.21E-04	-0.39	8.34E-03	1.08E-03	-0.33	4.36E-03
Q2M296	Methenyltetrahydrofolate synthase domain-containing protein	MTHFSD	2	0	1	1	4.45E-02	-0.07	1.41E-01	2.05E-01	-0.23	3.12E-01	6.87E-02	-0.10	1.12E-01
P01834	Ig kappa chain C region	IGKC	0	0	0	0	1.24E-01	-0.08	2.67E-01	2.54E-02	-0.37	7.37E-02	1.92E-06	-0.50	5.80E-05
P08603	Complement factor H	CFH	0	0	0	0	4.55E-03	-0.08	5.09E-02	3.27E-01	-0.07	4.34E-01	1.28E-04	0.27	1.04E-03
O95445	Apolipoprotein M	APOM	0	0	0	0	3.57E-01	-0.08	5.11E-01	1.22E-03	0.49	1.13E-02	5.78E-05	0.64	7.44E-04
P01009	Alpha-1-antitrypsin	SERPINA1	0	0	0	0	5.78E-01	-0.09	7.19E-01	5.14E-02	-0.43	1.22E-01	9.36E-01	-0.12	9.55E-01
P25311	Zinc-alpha-2-glycoprotein	AZGP1	0	0	0	0	6.34E-01	-0.09	7.47E-01	5.98E-02	0.28	1.39E-01	6.34E-04	-0.17	2.84E-03
P02774	Vitamin D-binding protein	GC	0	0	0	0	9.08E-02	-0.09	2.15E-01	8.34E-01	0.06	8.81E-01	5.41E-01	0.09	6.30E-01
P02768	Serum albumin	ALB	0	0	0	0	1.21E-02	-0.10	6.78E-02	7.61E-03	-0.10	3.72E-02	3.14E-05	-0.32	4.75E-04
P01008	Antithrombin-III	SERPINC1	0	0	0	0	2.19E-01	-0.10	3.84E-01	4.95E-01	-0.13	5.73E-01	7.75E-02	0.02	1.23E-01
P0DP01	Ig heavy chain V-I region HG3	IGHV1-3	0	0	0	0	1.39E-01	-0.11	2.84E-01	7.68E-04	-0.37	9.08E-03	1.92E-02	-0.55	3.71E-02
A0A0C4DH25		IGKV3D-20	0	0	1	0	6.67E-01	-0.13	7.66E-01	1.17E-02	-0.47	4.90E-02	1.65E-02	-0.31	3.38E-02
P0DOY2			0	0	0	0	1.41E-01	-0.13	2.84E-01	8.60E-03	-0.54	3.99E-02	1.83E-03	-0.32	6.42E-03
P0DP04			2	1	2	2	5.30E-01	-0.14	6.79E-01	NA	NA	NA	NA	NA	NA
P01023	Alpha-2-macroglobulin	A2M	0	0	0	0	2.31E-01	-0.15	3.90E-01	9.35E-01	0.00	9.50E-01	7.09E-01	0.08	7.80E-01
P01871	Ig mu chain C region	IGHM	0	0	0	0	1.55E-01	-0.15	2.98E-01	3.98E-01	0.18	4.84E-01	2.14E-05	1.05	3.70E-04
P02765	Alpha-2-HS-glycoprotein	AHSG	0	0	0	0	2.44E-01	-0.16	3.90E-01	2.32E-02	-0.49	7.25E-02	1.23E-01	-0.32	1.79E-01

P04217	Alpha-1B-glycoprotein	A1BG	0	0	0	0	2.26E-05	-0.16	1.64E-03	1.95E-01	-0.10	3.01E-01	1.31E-03	0.14	5.02E-03
P01624	Ig kappa chain V-III region POM		0	0	0	0	1.05E-02	-0.17	6.30E-02	8.76E-02	-0.44	1.68E-01	1.41E-05	-0.39	2.85E-04
B9A064	Immunoglobulin lambda-like polypeptide 5	IGLL5	0	0	0	0	1.43E-01	-0.18	2.84E-01	4.70E-01	-0.17	5.56E-01	8.43E-04	-0.52	3.52E-03
P06396	Gelsolin	GSN	0	0	0	0	3.07E-01	-0.19	4.60E-01	6.47E-02	-0.30	1.44E-01	4.94E-03	-0.26	1.40E-02
P02787	Serotransferrin	TF	0	0	0	0	1.61E-02	-0.19	7.74E-02	7.18E-02	-0.14	1.51E-01	2.94E-03	-0.31	9.30E-03
P80748	Ig lambda chain V-III region LOI	IGLV3-9	0	0	0	0	6.75E-02	-0.19	1.73E-01	2.79E-01	-0.31	3.95E-01	1.31E-04	-0.46	1.04E-03
A0A0B4J1V0		IGHV3-15	0	0	0	0	1.43E-01	-0.20	2.84E-01	3.93E-01	-0.23	4.84E-01	2.43E-03	-0.37	7.96E-03
A0A075B6P5	Ig kappa chain V-II region FR	IGKV2D-28	0	0	0	0	5.46E-03	-0.20	5.23E-02	7.66E-02	-0.33	1.56E-01	5.25E-04	-0.49	2.54E-03
P01857	Ig gamma-1 chain C region	IGHG1	0	0	0	0	2.59E-01	-0.21	4.03E-01	9.27E-02	-0.41	1.72E-01	3.89E-04	-0.69	1.96E-03
A0A075B6K4		IGLV3-10	1	0	0	0	2.27E-01	-0.21	3.90E-01	4.32E-02	-0.28	1.08E-01	2.97E-02	-0.87	5.29E-02
P19827	Inter-alpha-trypsin inhibitor heavy chain H1	ITIH1	0	0	0	0	4.46E-02	-0.21	1.41E-01	6.36E-01	-0.06	6.89E-01	9.81E-01	0.07	9.81E-01
Q96PD5	N-acetylmuramoyl-L-alanine amidase	PGLYRP2	0	0	1	0	1.27E-02	-0.22	6.80E-02	7.89E-01	0.17	8.41E-01	8.90E-01	0.25	9.28E-01
P01861	Ig gamma-4 chain C region	IGHG4	0	0	0	0	6.26E-03	-0.23	5.23E-02	5.34E-04	-0.37	8.34E-03	1.37E-03	-0.29	5.02E-03
P01860	Ig gamma-3 chain C region	IGHG3	0	0	0	0	2.93E-02	-0.23	1.09E-01	1.37E-02	-0.44	5.40E-02	4.01E-03	-0.43	1.18E-02
P10720	Platelet factor 4 variant	PF4V1	4	1	2	2	5.87E-01	-0.24	7.22E-01	NA	NA	NA	NA	NA	NA
P01619	Ig kappa chain V-III region B6		0	0	0	0	1.63E-02	-0.26	7.74E-02	7.68E-02	-0.58	1.56E-01	1.20E-02	-0.40	2.69E-02
P02751	Fibronectin	FN1	0	0	0	0	2.03E-02	-0.27	8.93E-02	1.90E-01	-0.15	3.00E-01	3.67E-04	0.41	1.93E-03
P43652	Afamin	AFM	0	0	0	0	1.06E-01	-0.29	2.40E-01	5.90E-01	0.04	6.50E-01	2.58E-02	-0.48	4.80E-02
A0A0C4DH31		IGHV1-18	1	0	1	1	5.13E-02	-0.29	1.50E-01	5.16E-02	-0.60	1.22E-01	9.68E-03	-0.74	2.25E-02
P06310	Ig kappa chain V-II region RPMI 6410	IGKV2D-30	0	0	0	0	2.66E-02	-0.30	1.02E-01	1.09E-01	-0.51	1.98E-01	3.25E-04	-0.40	1.87E-03
P02760	Protein AMBP	AMBP	0	0	0	0	7.13E-03	-0.30	5.23E-02	2.29E-02	-0.38	7.25E-02	5.93E-03	0.10	1.59E-02
P01602	Ig kappa chain V-I region HK102	IGKV1-5	2	0	0	1	1.57E-03	-0.33	2.69E-02	4.98E-01	-0.23	5.73E-01	7.42E-05	-0.56	8.16E-04
Q15582	Transforming growth factor-beta-induced protein ig-h3	TGFBI	0	0	1	0	9.58E-02	-0.35	2.22E-01	1.78E-02	-0.29	6.44E-02	5.38E-01	-0.15	6.30E-01
P01034	Cystatin-C	CST3	1	1	1	2	5.25E-02	-0.40	1.50E-01	5.71E-01	0.13	6.34E-01	NA	NA	NA
P19823	Inter-alpha-trypsin inhibitor heavy chain H2	ITIH2	0	0	0	0	1.04E-02	-0.42	6.30E-02	2.55E-02	-0.27	7.37E-02	9.47E-01	0.07	9.55E-01
P02775	Platelet basic protein	PPBP	0	0	0	0	4.02E-01	-0.43	5.49E-01	2.88E-01	-0.17	3.98E-01	1.56E-02	-0.59	3.36E-02

A0A0B4J1 V1	Ig heavy chain V-III region JON	IGHV3-21	0	0	0	0	1.08E-02	-0.46	6.30E-02	3.87E-01	0.18	4.84E-01	2.39E-02	-0.36	4.52E-02
P02771	Alpha-fetoprotein	AFP	0	0	0	0	3.63E-02	-0.46	1.31E-01	2.72E-02	-0.54	7.68E-02	1.38E-04	-1.30	1.04E-03
P01594	Ig kappa chain V-I region AU		1	0	0	0	1.39E-01	-0.46	2.84E-01	8.76E-05	-0.55	5.19E-03	3.52E-02	-0.41	6.17E-02
P04275	von Willebrand factor	VWF	0	0	0	0	7.56E-03	-0.47	5.23E-02	5.46E-04	-0.88	8.34E-03	1.68E-01	-0.82	2.26E-01
P69891	Hemoglobin subunit gamma-1	HBG1	1	0	1	2	5.96E-01	-0.51	7.26E-01	3.98E-01	1.19	4.84E-01	NA	NA	NA
P01705	Ig lambda chain V-II region NEI		5	1	0	0	7.66E-03	-0.56	5.23E-02	NA	NA	NA	3.98E-02	-0.49	6.79E-02
P27169	Serum paraoxonase/arylesterase 1	PON1	0	0	0	0	6.75E-02	-0.62	1.73E-01	3.97E-01	-0.05	4.84E-01	6.91E-01	0.09	7.67E-01
P60709	Actin, cytoplasmic 1	ACTB	0	0	0	0	1.89E-01	-0.70	3.46E-01	9.84E-01	0.24	9.84E-01	4.99E-03	-1.20	1.40E-02
P04040	Catalase	CAT	4	1	0	2	7.48E-01	-0.79	8.21E-01	NA	NA	NA	NA	NA	NA
P69905	Hemoglobin subunit alpha	HBA1	0	0	0	0	3.53E-01	-0.93	5.11E-01	4.61E-01	0.91	5.50E-01	3.00E-01	-0.29	3.78E-01
P69892	Hemoglobin subunit gamma-2	HBG2	0	0	0	0	4.33E-01	-0.94	5.79E-01	4.54E-01	0.84	5.46E-01	4.45E-01	-0.35	5.50E-01
P62328	Thymosin beta-4	TMSB4X	0	0	0	0	3.30E-01	-0.98	4.84E-01	1.63E-01	-0.69	2.76E-01	1.31E-01	-1.19	1.89E-01
P59666	Neutrophil defensin 3	DEFA3	0	1	0	1	3.79E-02	-1.06	1.33E-01	1.53E-02	-1.31	5.84E-02	6.71E-04	-2.92	2.90E-03
P21333	Filamin-A	FLNA	0	0	1	1	2.66E-02	-1.28	1.02E-01	8.59E-01	0.15	8.86E-01	1.57E-01	-1.08	2.14E-01
P68871	Hemoglobin subunit beta	HBB	0	0	0	0	2.07E-01	-1.38	3.68E-01	5.34E-01	0.82	6.08E-01	5.67E-01	-0.35	6.54E-01
P32119	Peroxiredoxin-2	PRDX2	1	0	2	2	1.54E-01	-1.73	2.98E-01	NA	NA	NA	NA	NA	NA
Q9HDC9	Adipocyte plasma membrane-associated protein	APMAP	6	1	1	1	NA	NA	NA	1.36E-02	0.53	5.40E-02	NA	NA	NA
P59998	Actin-related protein 2/3 complex subunit 4	ARPC4	7	2	4	1	NA	NA	NA	NA	NA	NA	NA	NA	NA
Q6PL18	ATPase family AAA domain-containing protein 2	ATAD2	5	1	3	3	NA	NA	NA	NA	NA	NA	NA	NA	NA
O75882	Attractin	ATRN	4	0	0	3	NA	NA	NA	9.65E-01	0.11	9.72E-01	NA	NA	NA
P30043	Flavin reductase (NADPH)	BLVRB	2	2	2	3	NA	NA	NA	NA	NA	NA	NA	NA	NA
P02745	Complement C1q subcomponent subunit A	C1QA	11	2	6	4	NA	NA	NA	NA	NA	NA	NA	NA	NA
P06681	Complement C2	C2	3	0	0	0	NA	NA	NA	9.09E-01	0.01	9.31E-01	8.92E-02	0.29	1.37E-01
P07360	Complement component C8 gamma chain	C8G	9	1	3	2	NA	NA	NA	NA	NA	NA	NA	NA	NA
P00915	Carbonic anhydrase 1	CA1	10	3	2	4	NA	NA	NA	NA	NA	NA	NA	NA	NA
O43866	CD5 antigen-like	CD5L	6	3	1	0	NA	NA	NA	NA	NA	NA	1.16E-05	0.98	2.81E-04

P00746	Complement factor D	CFD	9	3	2	2	NA	NA	NA	NA	NA	NA	NA	NA	NA
P36980	Complement factor H-related protein 2	CFHR2	6	0	0	4	NA	NA	NA	3.23E-01	0.39	4.32E-01	NA	NA	NA
P02452	Collagen alpha-1(I) chain	COL1A1	5	2	0	1	NA	NA	NA	2.44E-01	0.20	3.48E-01	8.72E-03	0.56	2.11E-02
P31146	Coronin-1A	CORO1A	10	4	3	3	NA	NA	NA	NA	NA	NA	NA	NA	NA
P12259	Coagulation factor V	F5	8	3	2	1	NA	NA	NA	NA	NA	NA	NA	NA	NA
P00740	Coagulation factor IX	F9	5	1	0	2	NA	NA	NA	NA	NA	NA	NA	NA	NA
P04406	Glyceraldehyde-3-phosphate dehydrogenase	GAPDH	8	3	3	4	NA	NA	NA	NA	NA	NA	NA	NA	NA
Q14520	Hyaluronan-binding protein 2	HABP2	3	0	0	0	NA	NA	NA	2.40E-02	0.64	7.25E-02	6.14E-05	0.97	7.44E-04
P02042	Hemoglobin subunit delta	HBD	10	4	3	5	NA	NA	NA	NA	NA	NA	NA	NA	NA
Q99878	Histone H2A type 1-J	HIST1H2AJ	7	3	5	4	NA	NA	NA	NA	NA	NA	NA	NA	NA
P62805	Histone H4	HIST1H4A	4	2	3	5	NA	NA	NA	NA	NA	NA	NA	NA	NA
P18065	Insulin-like growth factor-binding protein 2	IGFBP2	9	2	3	5	NA	NA	NA	NA	NA	NA	NA	NA	NA
P01772	Ig heavy chain V-III region KOL	IGHV3-66	7	5	3	3	NA	NA	NA	NA	NA	NA	NA	NA	NA
A0A0B4J1X5		IGHV3-74	4	3	1	3	NA	NA	NA	1.20E-01	-0.47	2.10E-01	NA	NA	NA
A0A0B4J1U7		IGHV6-1	11	5	5	2	NA	NA	NA	NA	NA	NA	NA	NA	NA
P01591	Immunoglobulin J chain	IGJ	5	1	0	1	NA	NA	NA	NA	NA	NA	1.67E-04	0.87	1.12E-03
A0A0C4DH72		IGKV1-6	4	1	0	2	NA	NA	NA	1.14E-01	-0.23	2.03E-01	NA	NA	NA
A0A075B6I4		IGLV10-54	4	1	0	2	NA	NA	NA	4.99E-02	-0.65	1.22E-01	NA	NA	NA
A0A075B6K0		IGLV3-16	6	1	1	1	NA	NA	NA	NA	NA	NA	7.36E-03	-1.25	1.89E-02
A0A075B6H9		IGLV4-69	10	1	4	2	NA	NA	NA	NA	NA	NA	NA	NA	NA
A0A075B6I9	Ig lambda chain V region 4A	IGLV7-46	7	1	1	3	NA	NA	NA	NA	NA	NA	NA	NA	NA
A0A075B6I0		IGLV8-61	8	3	3	0	NA	NA	NA	NA	NA	NA	8.63E-01	0.44	9.08E-01
P07195	L-lactate dehydrogenase B chain	LDHB	6	4	2	5	NA	NA	NA	NA	NA	NA	NA	NA	NA
P48357	Leptin receptor	LEPR	7	2	1	1	NA	NA	NA	2.22E-01	-0.18	3.24E-01	NA	NA	NA
P08519	Apolipoprotein(a)	LPA	9	3	2	0	NA	NA	NA	NA	NA	NA	NA	NA	NA

P02750	Leucine-rich alpha-2-glycoprotein	LRG1	5	2	1	5	NA	NA	NA	1.91E-01	0.89	3.00E-01	NA	NA	NA
P02788	Lactotransferrin	LTF	4	4	3	5	NA	NA	NA	NA	NA	NA	NA	NA	NA
O43196	MutS protein homolog 5	MSH5	9	4	4	4	NA	NA	NA	NA	NA	NA	NA	NA	NA
Q6UXB8	Peptidase inhibitor 16	PI16	3	2	0	1	NA	NA	NA	4.32E-04	-0.84	8.34E-03	1.54E-01	-0.19	2.11E-01
P62937	Peptidyl-prolyl cis-trans isomerase A	PPIA	9	2	5	6	NA	NA	NA	NA	NA	NA	NA	NA	NA
P21453	Sphingosine 1-phosphate receptor 1	S1PR1	6	0	2	2	NA	NA	NA	NA	NA	NA	NA	NA	NA
P05546	Heparin cofactor 2	SERPIN D1	12	4	3	5	NA	NA	NA	NA	NA	NA	NA	NA	NA
P05155	Plasma protease C1 inhibitor	SERPIN G1	6	2	1	0	NA	NA	NA	1.81E-03	0.55	1.47E-02	6.72E-03	0.55	1.77E-02
P02730	Band 3 anion transport protein	SLC4A1	7	2	3	5	NA	NA	NA	NA	NA	NA	NA	NA	NA
P02549	Spectrin alpha chain, erythrocytic 1	SPTA1	13	4	2	3	NA	NA	NA	NA	NA	NA	NA	NA	NA
Q9H254	Spectrin beta chain, non-erythrocytic 4	SPTBN4	13	5	3	5	NA	NA	NA	NA	NA	NA	NA	NA	NA
P49755	Transmembrane emp24 domain-containing protein 10	TMED10	9	2	4	3	NA	NA	NA	NA	NA	NA	NA	NA	NA
P63104	14-3-3 protein zeta/delta	YWHAZ	2	2	2	2	NA	NA	NA	NA	NA	NA	NA	NA	NA
P01701	Ig lambda chain V-I region NEW		3	1	1	1	NA	NA	NA	6.65E-02	1.07	1.44E-01	2.37E-01	-0.06	3.05E-01
P01703	Ig lambda chain V-I region NEWM		4	1	0	0	NA	NA	NA	NA	NA	NA	3.87E-02	-0.27	6.69E-02
P01704	Ig lambda chain V-II region TOG		5	3	2	2	NA	NA	NA	NA	NA	NA	NA	NA	NA
P01706	Ig lambda chain V-II region BOH		10	5	2	2	NA	NA	NA	NA	NA	NA	NA	NA	NA
P01709	Ig lambda chain V-II region MGC		6	1	1	3	NA	NA	NA	NA	NA	NA	NA	NA	NA
P01714	Ig lambda chain V-III region SH		8	3	2	2	NA	NA	NA	NA	NA	NA	NA	NA	NA

Supplementary Table 8. All proteins in the validation cohort sorted by DOL1 fold change. FC: log2(DOLX vs DOL0 fold change difference)

**Feature**	**DOL1 vs DOL0**		**DOL3 vs DOL0**		**DOL7 vs DOL0**	
	Log2(FC)	*q-value*	*Log2(FC)*	*q-value*	*Log2(FC)*	*q-value*
Cortisol lipid	-1.01	4.83E-01	-2.36	1.91E-02	-4.53	1.35E-04
IL-6	-0.38	4.25E-01	-1.98	1.52E-02	-1.98	8.70E-02
IL-10	-2.19	3.67E-01	-2.29	4.55E-02	-2.28	2.47E-02

Supplementary Table 9. Levels of cortisol lipid in the main cohort, as determined by metabolomics, and interleukin (IL) 6 and 10 as determined by a Cytokine Human Magnetic Panel bead array. FC: median fold-change difference relative to DOL0.

Protein Name	GN	DOL1	DOL3	DOL7	Protein Level	UPID
Alpha-2-macroglobulin	A2M	*-9.8%*	*0.0%*	*5.8%*		P01023
C4b-binding protein alpha chain	C4BPA	97.6%	*102.9%*	163.3%		P04003
Complement C1q subcomponent B	C1QB	*11.5%*	12.1%	*35.8%*		P02746
Complement C1q subcomponent C	C1QC	15.9%	50.4%	**51.8%**		P02747
Complement C1r subcomponent	C1R	*12.1%*	*22.4%*	36.9%		P00736
Complement C1s subcomponent	C1S	**32.2%**	31.6%	**28.4%**		P09871
Complement C2	C2		*0.4%*	*22.5%*		P06681
Complement C3	C3	*2.4%*	*7.7%*	*12.1%*		P01024
Complement C4-B	C4B	10.2%	33.1%	50.4%		P0C0L5
Complement C5	C5	*12.6%*	33.3%	38.7%		P01031
Complement component C6	C6	17.8%	*9.5%*	*17.4%*		P13671
Complement component C7	C7	*2.7%*	-21.3%	**-27.8%**		P10643
Complement component C8 alpha	C8A	52.0%	160.8%	**102.7%**		P07357
Complement component C8 beta	C8B	*22.5%*	77.8%	107.1%		P07358
Complement component C9	C9	**162.3%**	*75.4%*	*134.5%*		P02748
Complement factor B	CFB	**29.4%**	49.0%	**69.3%**		P00751
Complement factor H	CFH	-5.4%	*-5.1%*	20.8%		P08603
Complement factor I	CFI	*8.6%*	*16.8%*	*3.3%*		P05156
Ficolin-3	FCN3	79.7%	*-25.3%*	*-28.1%*		O75636
Plasma protease C1 inhibitor	SERPING1		46.8%	**46.9%**		P05155
Properdin	CFP	*1.6%*	*-21.5%*	*-28.9%*		P27918
Prothrombin	F2	*5.2%*	*-2.2%*	17.7%		P00734
Vitamin K-dependent protein S	PROS1	**56.2%**	27.3%	78.1%		P07225

Supplementary Table 10. Change of central complement system proteins in the validation cohort, at DOL-1, 3, and 7, relative to at birth (DOL0), i.e. 0% equals no change. Grey italic: p-value > 0.05, black: p-value < 0.05, black bold: q-value < 0.05. Trend marker red(blue): positive(negative) change compared to DOL0 (black).
